# Heterometallic Multinuclear Ruthenium Complexes as Cytotoxic Agents

**DOI:** 10.3390/biomedicines14051028

**Published:** 2026-04-30

**Authors:** Irena Kostova

**Affiliations:** Department of Chemistry, Faculty of Pharmacy, Medical University, 2 Dunav St., 1000 Sofia, Bulgaria; irenakostova@yahoo.com or i.kostova@pharmfac.mu-sofia.bg

**Keywords:** ruthenium-based complexes, heterometallic, multinuclear, cytotoxic agents

## Abstract

The design of multitargeted drug candidates has recently emerged as a highly attractive area of research. Numerous heterometallic compounds have been developed to enhance both the biological efficacy and physicochemical properties of monometallic metallodrugs. Combining classical transition metals with established antitumor activity, such as Pt, Ru, and Au, with other metal-based fragments offers the potential to generate complex compounds with improved pharmacokinetic and pharmacodynamic profiles. Incorporating different bioactive metal cations within a single molecular framework may enhance anticancer activity through metal-specific interactions with distinct biological targets or through improved physicochemical characteristics of the resulting heteronuclear complexes. Recent studies have underscored the significant progress and promising impact of this multitargeted strategy, particularly in systems that combine ruthenium with other biologically active metal centers. This approach may enable selective biological targeting and help overcome drug resistance. This review compiles and analyzes reported ruthenium-based heteronuclear complexes, offering a comprehensive and critical assessment of recent advances in the rational design and synthesis of novel multinuclear compounds as potential chemotherapeutic agents. Particular emphasis is placed on understanding structure–activity relationships, mechanistic pathways, and the role of metal–metal and metal–ligand interactions in modulating biological responses. The findings summarized herein highlight the remarkable efficacy of a wide range of multinuclear ruthenium anticancer complexes and support the hypothesis that synergistic and/or cooperative interactions between distinct metal-based fragments can significantly enhance pharmacological performance, including improved selectivity, stability, and cellular uptake. Furthermore, emerging insights into their modes of action, resistance profiles, and potential for targeted delivery underscore their promise as viable alternatives to conventional therapies. Overall, this dynamic and rapidly evolving field is poised to inspire continued interdisciplinary research and drive the development of next-generation metallodrugs with improved therapeutic indices and clinical potential.

## 1. Introduction

Over the past decades, interest in biologically active drug candidates, including heterometallic complexes, has increased considerably. This is particularly true for highly challenging diseases such as malignancies, in which multiple parallel pathways may operate simultaneously or where resistance to chemotherapy develops [1]. Studies on promising chemotherapeutic agents have paved the way for strategic approaches that combine multitarget fragments within a single molecular structure. Thus, the incorporation of bioactive targeting ligands [2] or different metallic fragments [3] into metal complexes offers new prospects for antitumor therapy.

In recent years, several general reviews on this topic have been published [4], focusing on heterometallic multinuclear compounds as theranostic agents [5,6,7]. These include polynuclear platinum-containing complexes [8], ferrocene-based multimetallic compounds [9], and other multinuclear complexes [10] developed for biomedical applications.

Transition metal complex compounds have been widely explored in medicinal chemistry, particularly in cancer therapy [11,12]. There is a clear need for novel metal-based antineoplastic agents with improved efficacy, selectivity and reduced toxicity compared with currently available anticancer drugs. In the search for alternative metal-based therapeutics, ruthenium complexes have attracted considerable attention as potential anticancer agents targeting various cellular pathways and have emerged as the most promising non-platinum candidates [13,14,15].

The growing prominence of platinum- and ruthenium-based complexes in oncology reflects a shift away from broadly toxic, non-selective chemotherapy toward more targeted, mechanistically nuanced treatments. Platinum drugs established the clinical value of metal-based chemotherapy, but their toxicity and resistance issues drove innovation. Ruthenium complexes—and next-generation platinum derivatives—are more selective, mechanistically diverse, and chemically tunable, aligning better with modern, precision-focused cancer treatment strategies. Among metal-based compounds, many ruthenium complexes have demonstrated lower toxicity and greater selectivity toward malignant cells than existing metal-based drugs, leading to a rapid expansion of their potential applications. Ruthenium complexes have also been shown to exhibit synergistic effects when combined with conventional chemotherapeutic agents [16]. Furthermore, these antineoplastic compounds have been extensively investigated as phototherapeutic and bioimaging agents [17].

In addition, ruthenium complexes can serve as tumor theranostic agents, integrating diagnosis and therapy for various cancers while reducing adverse effects [18,19,20]. A number of Ru-based complexes have displayed potent antiproliferative activity in both in vitro and in vivo models, representing attractive alternatives to existing metal-based anticancer drugs. Importantly, they can overcome major limitations associated with traditional agents, such as poor tumor selectivity and significant toxicity to normal cells.

The advantages of ruthenium over platinum include its wider range of accessible oxidation states, relatively slow ligand-exchange kinetics, enhanced cellular uptake, lower toxicity, reduced likelihood of drug resistance, and its ability to mimic iron, which facilitates binding to biological molecules such as proteins.

Under physiological conditions, ruthenium typically exists in two stable oxidation states, Ru(II) and Ru(III), with the former generally exhibiting higher reactivity [21,22]. Ru(II), which has a d^6^ electronic configuration, is diamagnetic, whereas Ru(III), with a d^5^ configuration, is paramagnetic. Owing to their favorable thermodynamic and kinetic stability and higher effective nuclear charge, Ru(III) complexes can act as prodrugs in hypoxic and acidic environments [23]. Both Ru(II) and Ru(III) cations display strong affinity for nitrogen- and sulfur-donor ligands. Their complexes commonly adopt an octahedral, hexacoordinated geometry, enabling participation in diverse redox processes [24]. These characteristics of ruthenium complexes are likely important contributors to their biological activity.

The octahedral coordination geometry of Ru(II) and Ru(III) complexes, in contrast to the square-planar geometry of Pt(II) complexes, enables distinct mechanisms of action and modes of reactivity compared with cisplatin [25,26]. Furthermore, relative to conventional platinum-based drugs, many ruthenium complexes exhibit greater aqueous solubility under physiological conditions, contributing to enhanced activity against platinum-resistant cancer cells [27]. This increased solubility also facilitates modulation of the hydrophilic–hydrophobic balance of Ru-based complexes, thereby promoting improved uptake by tumor cells [28].

The structurally similar mononuclear Ru(III) complexes NAMI-A and KP1019 have demonstrated remarkable anticancer potential, exhibiting distinct in vitro and in vivo activities. NAMI-A is primarily associated with antiangiogenic and antimetastatic effects in secondary tumors, whereas KP1019 shows greater activity against primary tumors [29]. Both compounds are capable of targeting DNA and proteins, displaying binding modes that differ from those of cisplatin and suggesting the involvement of multiple mechanisms of action. Under physiological conditions, they can be reduced to the more reactive Ru(II) species and are therefore considered prodrugs [30]. The water-soluble version of KP1019, KP1339, has completed a clinical phase I trial for the treatment of neuroendocrine tumors. Its sodium salt form, NKP1339, is regarded as a promising clinical candidate due to its enhanced water solubility, which enables administration at higher doses. TLD1433 is another promising agent, particularly for bladder cancer treatment. Together with other polypyridyl Ru(II) derivatives, such as photoactive Ru(bpy)_3_^2+^, it has shown considerable potential in photodynamic therapy (PDT) and DNA binding applications [31,32]. Additionally, organometallic arene-based half-sandwich ruthenium complexes, including RAPTA, RM175, and ONCO4417, have also received substantial consideration for anticancer applications, demonstrating notable activity both in vivo (e.g., RAPTA) and in vitro (e.g., RM175) [31].

Ruthenium complexes were initially expected to inhibit tumor growth through interactions with cellular DNA, in a manner similar to cisplatin. However, these complexes exhibit several important differences compared with cisplatin [33,34]; for example, Ru complexes tend to accumulate preferentially in neoplastic tissues rather than in normal tissues. In many cases, they demonstrate greater efficacy against metastatic lesions than against primary tumors. Taken together, these observations suggest that ruthenium complexes may exert cytotoxic and antineoplastic effects through mechanisms distinct from those of cisplatin and other platinum-based agents [35,36,37,38]. Consequently, ruthenium complexes have been regarded as promising alternatives to platinum-based drugs. Many ruthenium complex and organometallic compounds display relatively low toxicity, and some have shown notable selectivity toward malignant cells [33]. These properties may be partly attributed to ruthenium’s ability to mimic iron in binding to biological macromolecules, including serum transferrin, albumin, and other proteins [34].

In general, the proposed mechanisms explaining why ruthenium complexes exhibit lower toxicity than platinum-based drugs include the reduction activation mechanism and the iron-mimicking hypothesis. The activation-by-reduction mechanism is based on the observation that Ru(III) complexes and related organometallic compounds are more inert than their corresponding Ru(II) species. Tumor cells typically possess a more reducing intracellular environment due to hypoxia (low oxygen levels) compared with normal cells [39,40]. As a result, many reported Ru(III) compounds cause negligible damage to healthy tissues, whereas in the hypoxic regions of malignant cells, Ru(III) is more readily reduced to the more reactive Ru(II) form [41]. This process depends on endogenous reducing agents such as ascorbate and glutathione, as well as the overall reducing conditions characteristic of hypoxic tumor microenvironments. The iron-mimicking hypothesis arises from the fact that iron and ruthenium belong to the same group of the periodic table, suggesting that ruthenium may imitate iron in its interactions with biological macromolecules. However, both hypotheses have faced significant criticism [42]. Furthermore, the mechanism by which ruthenium complexes enter cells remains a subject of ongoing debate [43].

The appeal of Ru complexes comes from a combination of tunable toxicity profiles and potentially improved tumor selectivity, but both properties are highly dependent on the structures. Their lower systemic toxicity, compared with platinum-based therapeutics is connected with their kinetic inertness, multiple oxidation states (Ru(III) complexes are often prodrugs) and selective accumulation in tumors. Depending on ligands, ruthenium complexes can selectively interact with DNA (less aggressive than platinum), mitochondria, kinases, thiol-rich enzymes, albumin, and transferrin, although this selectivity is usually partial, not absolute. The current research strategies to improve both toxicity and specificity include the development of targeted delivery systems, photoactivation, fine-tuning hydrophobicity and redox potential and particularly design of dual-action complexes [41,42,43].

Although a wide range of mononuclear Ru-based complexes has been developed, the emergence of multinuclear ruthenium complexes as bioactive compounds has attracted considerable attention. Polynuclear Ru complexes, which contain more than one metal cation, are particularly appealing in medicinal chemistry because they exhibit distinctive properties compared with their mononuclear counterparts, including diverse charge states, redox reactivity, and enhanced selectivity toward various biomolecules [44,45,46,47]. The question about the half-life time of heterometallic multinuclear ruthenium complexes under physiological conditions (37 °C, pH ~7.4) is also of great importance. Their half-life (t½) varies widely depending on the oxidation state, nuclearity (dinuclear, trinuclear, etc.), bridging ligands (pyrazine, bipyridine, peptide linkers), charge, hydrophilicity and target environment. In general, the multinuclear ruthenium complexes are moderately stable in blood but reactive in cells. Many heterometallic systems remain intact to >24 h in aqueous and physiological media (highly stabilized metalla-assemblies or arene-bridged structures), but some labile ligand systems show ligand exchange over several hours [46,47].

Like other heteronuclear systems, Ru-based heterometallic complexes have been investigated for their antitumor activity through association with different metal centers. These complexes are designed as single molecular entities to enhance pharmacological performance. Compared with their mononuclear counterparts [48,49,50], they have shown several advantages as promising anticancer agents.

Enhanced cytotoxicity, stronger DNA-binding affinity, improved selectivity, and the ability to overcome drug resistance have frequently been reported for heteronuclear complexes containing multiple Ru and other metal centers [51,52], demonstrating clear benefits over their mononuclear precursors. Therefore, heterometallic multinuclear ruthenium complexes synergistically integrate the intrinsic properties of distinct metal-based moieties within a single structure, thereby enhancing their overall therapeutic potential. Their properties can be further fine-tuned through the careful selection of metal combinations, bridging ligands, and co-ligands to maximize synergistic effects.

This review highlights the potential of multinuclear heterometallic ruthenium-based drug candidates, placing them in context with individual metal fragments and established chemotherapeutics. The discussed complexes exhibit remarkable diversity, not only in their metal centers and combinations but also in the biologically active organic ligands they incorporate. This overview collects numerous heteronuclear Ru-containing compounds, examining their design strategies and comparing cytotoxic profiles, mechanistic contributions, and anticancer activities linked to the metal centers. Emphasis is placed on the promise of heterometallic ruthenium complexes as next-generation cancer therapeutics.

The search strategy was applied to various electronic databases, including Scopus, Web of Science, PubMed, and Google Scholar, which index peer-reviewed, high-quality scientific biomedical and pharmaceutical literature. The searches used the terms “Ruthenium”, “Heteronuclear Complexes” and “Anticancer Drugs” and included various related keywords in order to be broad enough, to avoid missing relevant citations and to ensure consistency of peer-reviewed content. All cited records were carefully screened to ensure the scientific rigor and status of the current review paper.

## 2. Ruthenium–Platinum Heteronuclear Complexes

Platinum compounds developed cancer therapy by forming DNA crosslinks that block replication and trigger apoptosis. However, their widespread use exposed major problems, such as severe side effects, development of drug resistance and lack of tumor selectivity. These issues created strong motivation to develop alternatives with better safety and efficacy profiles.

Among numerous transition elements, ruthenium cations and Ru-based complexes have emerged as promising alternatives to platinum drugs, offering comparable or superior therapeutic efficacy with lower toxicity. Ruthenium complexes act through multiple mechanisms and target a wide range of cellular components. They offer several advantages over platinum drugs, including reduced toxicity, improved effectiveness, lower drug resistance, and enhanced cellular permeation. Ruthenium-based drugs often exist in multiple oxidation states (Ru(II)/Ru(III)), allowing activation specifically in the hypoxic environment typical of tumors. Additionally, they can mimic iron and bind to biomolecules, enhancing uptake in rapidly dividing cancer cells. many of them have shown promise in clinical trials, particularly for metastatic cancers.

Recently, the development of ruthenium–platinum heterometallic complexes has opened new avenues for designing more effective antineoplastic agents. Bifunctional Pt–Ru complexes combining cytotoxic and antimetastatic properties are a particularly promising area. Most of the reported heterometallic ruthenium–platinum complexes demonstrate multifunctional interactions with nuclear DNA, mitochondrial DNA, RNA, and proteins, enabling their use in anticancer therapy, photodynamic therapy, diabetes treatment, and as molecular sensors. Compared to cisplatin and their respective Ru-based monometallic precursors, numerous Ru–Pt complexes exhibit enhanced cytotoxicity and cancer selectivity. By covalently binding cisplatin to DNA, photoactive Ru(II)–Pt(II) complexes can localize photodynamic therapy agents precisely at the site of action. The design and biomedical applications of ruthenium- and platinum-based mono- and mixed-metal complexes have been recently reviewed [53].

Heterometallic Ru(II)–Pt(II) complexes combine remarkable physicochemical properties with unique mechanisms of action, yielding distinctive biological activity profiles [54,55,56]. In particular, Ru(II)–polypyridyl chromophores integrated with DNA-targeting Pt(II) centers exhibit outstanding electrochemical and spectroscopic behavior, alongside potent photodynamic therapy (PDT) efficacy. Sakai and co-workers have reported the complex [Ru(bpy)_2_{μ-bpy(CONH-(CH_2_)-NH_2_)_2_}PtCl_2_]^2+^ where bpy = 2,2′-bipyridine, that exhibited characteristic photophysical properties and was capable of cleaving pBR322 DNA upon visible light irradiation under air-saturated conditions [57]. Many heterometallic Pt–Ru complexes of the form [(bipy)_2_Ru(BL)PtCl_2_]^2+^ (BL = bridging ligand) have been synthesized, including those with 2,3-bis(2-pyridyl)pyrazine (2,3-dpp) bridge [58], 2,3-bis(2-pyridyl)quinoxaline (dpq), and bis(2-pyridyl)benzoquinoxaline (dpb) ligands [59]. Subsequent studies investigated the DNA interactions of these complexes, suggesting a cisplatin-like non-covalent binding mode [60]. Further work using the same bridging ligands (dpp, dpq, dpb) but with 2,2′:6′,2″-terpyridine (tpy) as ancillary ligands yielded a series of compounds that were shown to feature tunable charge transfer bands based on the stability of the π* orbitals on the bridging ligand, along with substantial DNA binding for all the bridging ligands [61]. Jain et al. have synthesized a Ru–Pt heterobimetallic complex [(tpy)RuCl(dpp)PtCl_2_]^+^ where tpy = 2,2′:6′,2″-terpyridine; dpp = 2,3-bis(2-pyridyl)pyrazine, which exhibits intriguing electrochemical and photophysical properties. This complex not only cleaves DNA upon visible-light irradiation but also demonstrates antibacterial activity [62]. The same group has also reported the complexes of the type [(Ph_2_phen)Ru(BL)PtCl_2_]^2+^ where BL = 2,3-bis(2-pyridyl)pyrazine (dpp) or 2,3-bis(2-pyridyl)quinoxaline (dpq), where incorporation of Ph_2_phen enhances spectroscopic and photophysical properties. In these complexes, the Pt site binds to pUC18 DNA, causing decreased DNA migration similar to cisplatin, while the Ru(II) center facilitates photocleavage of pUC18 DNA via an oxygen-dependent mechanism [63]. Schilden et al. investigated a Ru–Pt bimetallic complex with a more flexible linker between the metal centers, specifically a diethyleneglycol unit [64]. The complex of the form [(tpy)Ru(dtdeg)PtCl]^3+^ (dtdeg = bis [4′-(2,2′:6′,2″-terpyridyl)]-diethyleneglycol ether) was shown to bind non-covalently to 9-ethylguanine. This behavior is consistent with the known non-covalent DNA binding of polypyridyl–Ru complexes, with the 2+ charge increasing target affinity, in combination with the cytotoxic effects of the Pt moiety [Pt(tpy)Cl]Cl.2H_2_O [65].

Ruthenium–platinum complexes represent a unique class of multifunctional anticancer agents that integrate both photochemically activated bioactivity (PAB) and thermally activated bioactivity (TAB) within a single molecular framework. In these complexes, the ruthenium center serves a dual role as a light absorber and a photochemical effector, while the cisplatin-like platinum moiety acts as a thermally activated cytotoxic unit through bridging ligands. This dual functionality enables synergistic activity, allowing the complexes to simultaneously induce oxidative stress via the Ru center and DNA platination via the Pt moiety. Such cooperative mechanisms position ruthenium–platinum complexes as promising candidates for advanced photodynamic therapy (PDT), offering multiple, complementary pathways for cancer cell eradication and potential strategies to overcome conventional drug resistance.

**Figure 1:**
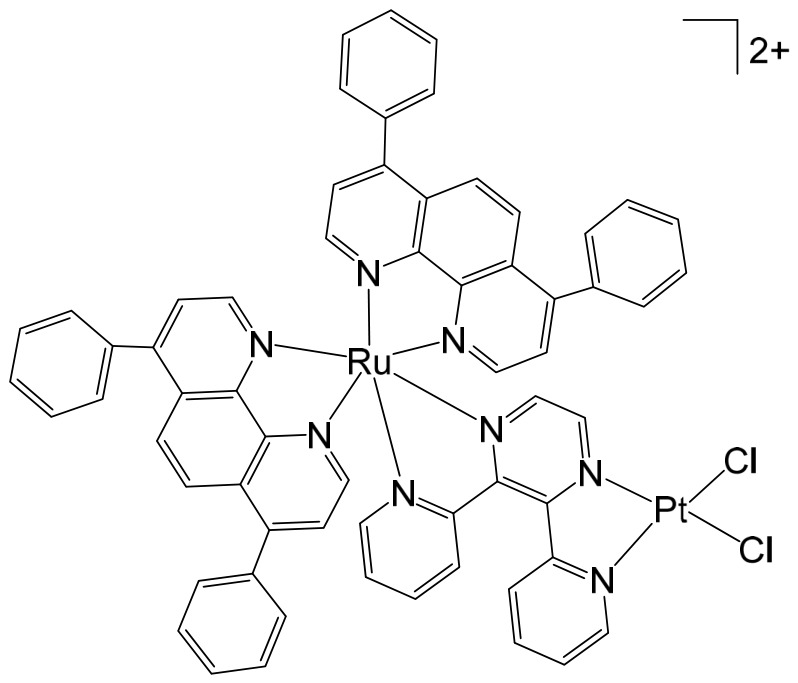
Structure of [(Ph_2_phen)_2_Ru(dpp)PtCl_2_]^2+^.

The supramolecular heterobimetallic complex [(Ph_2_phen)_2_Ru(dpp)PtCl_2_]^2+^ (Figure 1), where Ph_2_phen = 4,7-diphenyl-1,10-phenanthroline; dpp = 2,3-bis(2-pyridyl) pyrazine, has emerged as a highly promising candidate for photochemically induced anticancer applications. This complex has demonstrated enhanced DNA modification capabilities, significant inhibition of cell proliferation, and potent antiproliferative activity specifically against F98 malignant glioma cells when exposed to visible light irradiation at 455 and 625 nm [66]. Notably, its activity under these conditions surpasses that of cisplatin, highlighting the potential of light-activated heterobimetallic complexes as more effective chemotherapeutic agents [66]. Zhu et al. further reported that this polyazine-bridged Pt(II)–Ru(II) complex exhibits pronounced cytotoxicity toward the tested cells, which is dramatically enhanced upon blue light irradiation, suggesting that the complex can act as a photo-activated cytotoxic agent with controllable activity. Building on these findings, researchers have synthesized a series of structurally diverse polyazine-bridged Ru(II)-Pt(II) tetrametallic supramolecules, including [{(Ph_2_phen)_2_Ru(dpp)}_2_Ru(dpp)PtCl_2_](PF_6_)_6_ and [{(Ph_2_phen)_2_Ru(dpp)}_2_Ru(dpq)PtCl_2_](PF_6_)_6_ (Ph_2_phen = 4,7-diphenyl-1,10-phenanthroline, dpp = 2,3-bis(2-pyridyl)pyrazine, dpq = 2,3-bis(2-pyridyl)quinoxaline). These tetrametallic assemblies, along with their corresponding trimetallic precursors, have been thoroughly investigated to understand their redox behavior, spectral characteristics, and photophysical properties [67]. The studies revealed that these complexes efficiently absorb light across both the UV and visible regions, displaying intense metal-to-ligand charge transfer (MLCT) transitions in the visible range around 540 nm. Such photophysical properties not only underpin their enhanced photoinduced cytotoxicity but also indicate potential applications in photodynamic therapy and as multifunctional supramolecular constructs for light-driven biomedical interventions.

Pt(II) complexes are widely used in anticancer therapy, but their clinical use is often limited by severe side effects. To address this, Pt(IV) complexes have been developed as prodrug candidates, which can be reduced intracellularly to the active Pt(II) species, thereby potentially reducing toxicity. In parallel, Ru(II) polypyridine complexes have emerged as effective photosensitizers for photodynamic therapy (PDT), owing to their high reactive oxygen species (ROS) generation, water solubility, and excellent photo- and chemical stability. To date, only a limited number of ruthenium–platinum polypyridyl complexes have been explored for their bioactivity as photosensitizing agents, with most studies focusing on heterometallic Ru(II)–Pt(II) complexes. Recently, Karges et al. reported novel Ru(II)–Pt(IV) conjugates that integrate chemotherapy and PDT, specifically designed to target drug-resistant cancers [68]. Upon cellular uptake, the Pt(IV) center is reduced to Pt(II), activating its cytotoxic potential. The ability of these Ru(II)–Pt(IV) conjugates to overcome drug resistance has been extensively studied in human ovarian carcinoma cell lines, including the cisplatin-resistant (A2780 cis) and doxorubicin-resistant (A2780 ADR) variants, as well as the parental A2780 line.

**Figure 2:**
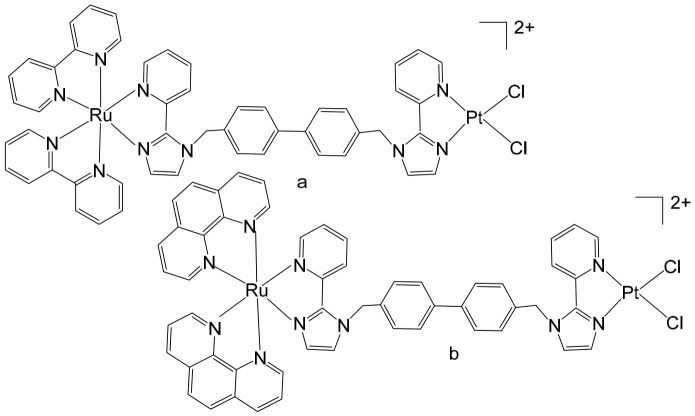
Ruthenium(II)–platinum(II) polypyridyl complexes of the type [Ru(bpy)_2_(BPIMBp)PtCl_2_]^2+^ (**a**) and [Ru(phen)_2_(-BPIMBp)PtCl_2_]^2+^ (**b**).

Heterobimetallic ruthenium(II)–platinum(II) polypyridyl complexes of the type [Ru(bpy)_2_(BPIMBp)PtCl_2_]^2+^ (Figure 2a) and [Ru(phen)_2_(-BPIMBp)PtCl_2_]^2+^ (Figure 2b) have been successfully synthesized using their corresponding precursors, [Ru(bpy)_2_BPIMBp]^2+^ and [Ru(phen)_2_BPIMBp]^2+^, where BPIMBp stands for 1,4′-Bis-{(2-pyridin-2-yl)-1H-imidazol-1-yl)methyl}-1,1′-biphenyl). The design of these complexes follows a heterobimetallic strategy, in which a Ru(II) polypyridyl unit, serving as a photosensitizer, is covalently linked to a cis-PtCl_2_ moiety, a well-known nucleotide covalent binder, via a rigid bridging ligand [69]. This dual-metal architecture is intended to combine the photophysical properties of the ruthenium center with the chemotherapeutic activity of platinum, potentially enabling a synergistic mechanism of action against cancer cells. The structural arrangement of the complexes is hypothesized to enhance their selectivity and efficacy, making them promising candidates for applications in photodynamic therapy (PDT). The underlying rationale is that each metallic center may operate via a distinct mechanism, allowing for complementary or additive effects while attenuating undesired side effects. To better understand their biological potential, the interactions of these complexes with CT-DNA were examined using a variety of biophysical and biochemical methods. Both intrinsic DNA cleavage in the absence of light (dark conditions) and photocleavage under blue visible light (~450 nm) were systematically evaluated. In addition, the ability of the complexes to induce autophagy, as well as their light-triggered cytotoxic effects on MCF-7 breast cancer cells, were investigated. The results demonstrated that these Pt(II)–Ru(II) heterobimetallic complexes exhibit strong phototoxic activity, highlighting their potential as effective photosensitizers in PDT and providing insights into the role of dual-metal coordination in enhancing anticancer activity [69,70,71].

**Figure 3:**
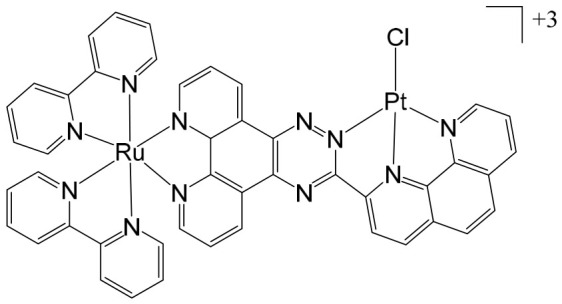
The chemical structure of chiral Λ/Δ-Pt(II)–Ru(II) complexes.

Among metal complexes, Pt(II) compounds have received extensive attention due to their distinctive square planar geometry, which allows for efficient and selective interactions with biomolecules such as DNA and proteins. This structural feature is particularly important in the context of anticancer therapeutics, as it enables precise targeting of nucleic acids and modulation of cellular pathways. One notable advancement in this area is the development of Pt(II)–Ru(II) complexes, which combine the cytotoxic potential of platinum with the photophysical properties of ruthenium to overcome limitations associated with traditional platinum-based drugs. For instance, a Pt(II)–Ru(II) complex designed by Zheng et al. demonstrated the ability to overcome cisplatin resistance by selectively inducing photodamage to mitochondrial DNA (mtDNA) [72]. The heterobimetallic complex [Ru(dip)_2_(μ-bpm)PtCl_2_]Cl_2_, where dip = 4,7-diphenyl-1,10-phenanthroline; bpm = 2,2′-bipyrimidine), along with its mononuclear analog [Ru(dip)_2_(bpm)]Cl_2_, have been synthesized and extensively characterized. This complex exhibits remarkable binding affinity toward mtDNA, inducing damage both in the absence of light and under visible light irradiation. Mechanistically, it disrupts mitochondrial function by reducing mtDNA amplification and copy number, altering transcriptional levels of mitochondria-encoded genes, and interfering with the organelle’s physiological processes. When exposed to light, it triggers caspase-dependent apoptosis, highlighting its potential as a light-activated anticancer agent. Notably, these complexes demonstrate low systemic toxicity while maintaining potent in vivo antineoplastic activity upon light irradiation, underscoring their therapeutic promise [72]. Beyond these examples, heteronuclear Pt(II)–Ru(II) complexes have been further explored for their capacity to selectively inhibit cancer cell proliferation. Xiong et al. reported the synthesis of novel chiral binuclear Λ/Δ-Pt(II)–Ru(II) complexes (Figure 3), which function as telomerase dysfunction-inducing chemotherapeutic agents [73]. The incorporation of the Ru(II) moiety not only confers chirality but also enhances the complexes’ binding specificity and affinity for negatively charged DNA structures. Among these enantiomers, the Δ-form exhibits a slightly stronger G-quadruplex stabilization effect and higher cytotoxicity in tumor cells compared to the Λ-form, highlighting the importance of stereochemistry in designing effective anticancer agents. Overall, these findings illustrate the versatility of Pt(II)–Ru(II) complexes, combining structural precision, light-activated cytotoxicity, and DNA-targeted selectivity. They represent a promising class of multifunctional metallodrugs capable of overcoming conventional chemotherapy resistance while minimizing off-target toxicity.

**Figure 4:**
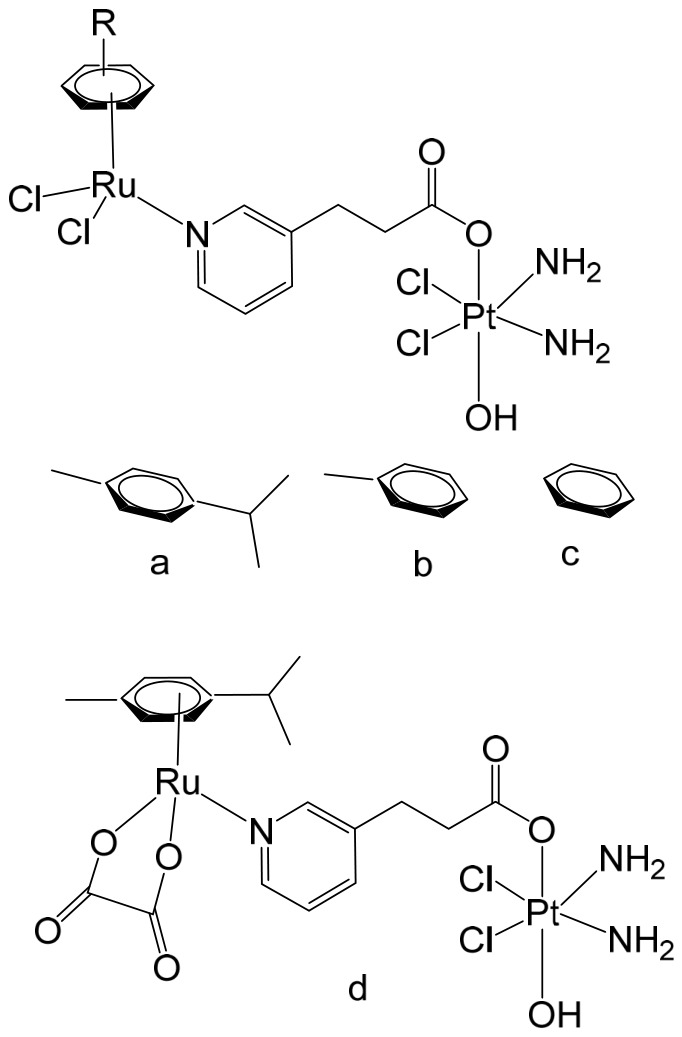
Water-soluble platinum(IV)–ruthenium(II) heterobinuclear complexes (**a**–**d**).

Ma et al. synthesized and fully characterized a series of four water-soluble platinum(IV)–ruthenium(II) heterobinuclear complexes (Figure 4a–d), collectively referred to as ‘‘ruthplatins”, with the objective of integrating the complementary pharmacological properties of both metal centers into a single bifunctional anticancer platform [74]. This rational design strategy was intended to exploit the well-established DNA-targeting capability of platinum drugs alongside the distinct coordination chemistry and biological versatility of ruthenium complexes, thereby addressing major limitations of conventional platinum-based chemotherapy, such as drug resistance and metastatic progression. Biological evaluation revealed that these bimetallic drug candidates effectively overcame cisplatin resistance and inhibited tumor cell migration. Remarkably, they demonstrated up to a two-order-of-magnitude enhancement in cytotoxic activity in cisplatin-resistant cell lines compared with cisplatin itself, indicating a fundamentally different and potentially synergistic mode of action. In addition to their potent antiproliferative effects, the compounds significantly impaired cancer cell migration, highlighting their antimetastatic potential. Structurally, ruthplatins (Figure 4a–c) incorporate η6-cymene ligands bearing different substituents on the ruthenium center, whereas the ruthplatin (Figure 4d) contains an oxalate ligand instead of the two chloride ligands typically coordinated to ruthenium. These structural variations were designed to modulate electronic properties, reactivity, and biological performance. Importantly, all four ruthplatins (Figure 4a–d) exhibited higher water solubility than cisplatin, a feature that may contribute to improved bioavailability and facilitate clinical translation. The complexes showed substantial cytotoxic activity across a broad panel of human cancer cell lines, including ovarian (A2780, A2780cisR), lung (A549, A549R), breast (MDA-MB-231, MCF-7), cervical (HeLa), and leukemia (HL60) models, with IC_50_ values generally below 5 μM. Notably, they displayed lower cytotoxicity toward normal MRC-5 lung fibroblasts compared with cisplatin, suggesting enhanced selectivity for malignant cells and a potentially improved therapeutic index. Among the series, complex (Figure 4a) emerged as the most potent compound. Mechanistic investigations indicated that these ruthplatins operate through pathways distinct from those of cisplatin, supporting the hypothesis that dual-metal coordination frameworks can generate unique biological responses. Ongoing studies are focused on elucidating their cellular uptake mechanisms, intracellular localization, biomolecular targets, and overall pharmacological behavior. Collectively, the formation of these heterobinuclear complexes resulted in enhanced cytotoxicity, improved activity against resistant cancer phenotypes, and significant antimetastatic effects compared with reference platinum-based drug candidates currently under clinical development. Together with their favorable aqueous solubility profile, these attributes underscore the promise of ruthplatins as advanced candidates for further preclinical investigation and potential clinical application.

**Figure 5:**
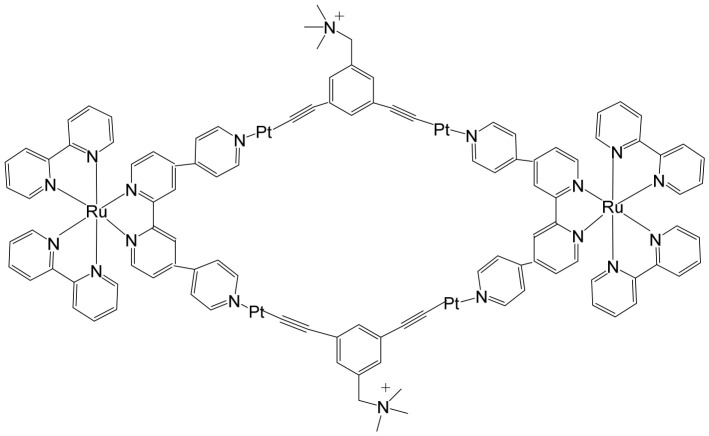
Structure of heterometallic Ru–Pt supramolecular metallacycle.

Over the past few decades, an increasingly diverse array of discrete supramolecular coordination complexes (SCCs) with precisely defined sizes, geometries, and tunable biological properties has been rationally designed and efficiently synthesized via coordination-driven self-assembly. This strategy exploits coordination interactions between Lewis-acidic metal-containing acceptors and Lewis-basic organic donors, enabling the predictable construction of architecturally complex systems with tailored functions. Zhou and co-workers reported the fabrication of a heterometallic Ru–Pt supramolecular metallacycle through this approach, combining the advantageous photophysical characteristics of Ru(II) polypyridyl complexes with the structural and functional features of Pt(II)-based SCCs [75]. In their design, a mononuclear Ru(II) building block bearing two terminal pyridyl nitrogen donor sites was coordinated with tetramethylammonium-modified bis(phosphine) Pt(II) acceptor clips, affording the heterometallic Ru–Pt metallacycle (Figure 5). This discrete assembly was evaluated as a two-photon photodynamic therapy (PDT) agent [75]. Comprehensive studies examined its photophysical behavior, singlet oxygen quantum yield, cellular uptake efficiency, and intracellular localization profile. Beyond preserving the intrinsic photostability and strong two-photon absorption characteristics of the Ru(II) polypyridyl precursor, incorporation into the metallacyclic framework led to several enhanced properties. The complex displayed markedly red-shifted emission extending into the near-infrared region, a significantly enlarged two-photon absorption cross-section, and improved singlet oxygen generation efficiency. Collectively, these enhancements broaden its functional scope and underscore its strong potential as an advanced photosensitizer for two-photon PDT applications. The heterometallic Ru–Pt metallocycle exhibited near-infrared (NIR) emission and an extended luminescence lifetime, rendering it particularly advantageous for bioimaging applications. These photophysical properties allow deep tissue penetration, reduced photodamage to healthy tissues, and minimal interference from light scattering and cellular autofluorescence, thereby enabling precise monitoring of cellular uptake and intracellular distribution. The cyclization-driven supramolecular assembly expanded the molecular orbitals of the Ru(II) donors onto the Pt centers, which enhanced spin–orbit coupling and promoted efficient intersystem crossing. At the same time, the rigidification of the Ru(II) framework suppressed nonradiative decay pathways. As a result, the metallacycle displayed a high quantum yield, comparable to other recently reported two-photon-active bioorganometallic systems [76,77,78,79]. As illustrated in Figure 5, the large, highly charged macrocyclic structure significantly facilitated cellular internalization, likely through electrostatic interactions with negatively charged cellular membranes. Cellular studies demonstrated selective dual localization in both mitochondria and nuclei, a feature that is particularly advantageous for photodynamic therapy (PDT). Upon light activation, the metallacycle generated singlet oxygen (^1^O_2_), which simultaneously disrupted mitochondrial membrane potential and induced oxidative damage to intranuclear DNA. This dual-targeting mechanism amplified therapeutic efficacy by attacking two critical cellular organelles, thereby triggering apoptosis and enhancing overall cytotoxic outcomes. Importantly, the metallacycle exhibited low dark toxicity, with IC_50_ values ranging from 65.2 to 80.9 μM, indicating good biocompatibility in the absence of irradiation. However, upon photoirradiation at 450 nm, substantial intracellular ^1^O_2_ generation led to markedly enhanced photocytotoxicity, with IC_50_ values of 0.71–4.4 μM. This pronounced difference between dark and light conditions underscores its strong photo-responsive behavior and therapeutic selectivity. In vivo evaluations in tumor-bearing mice further confirmed its therapeutic potential. Under low-dose 800 nm irradiation, the metallacycle effectively suppressed tumor growth with minimal systemic toxicity and negligible side effects. Two-photon PDT studies demonstrated efficient ablation of malignant tissues, benefiting from deeper tissue penetration and spatially confined activation. Collectively, these findings highlight the metallacycle as a promising, safe, and highly efficient two-photon PDT agent with strong potential for future clinical translation.

Self-assembled metallacycles and metallacages have attracted increasing attention as advanced photosensitizers for photodynamic therapy (PDT) in cancer treatment due to their tunable structures, enhanced photophysical properties, and multifunctionality. By integrating mononuclear Ru(II)-based photosensitizers with complementary di-Pt(II) or tri-Pt(II) acceptors through coordination-driven self-assembly, well-defined supramolecular architectures can be constructed, including [2 + 2] rhomboidal metallocycles and [6 + 4] octahedral metallacages. These discrete assemblies benefit from improved stability, controlled geometry, and synergistic photodynamic performance arising from the incorporation of multiple photoactive units within a single framework. Zhou and co-workers reported the rational design and synthesis of a Ru–Pt bimetallic octahedral metallacage as an efficient photosensitizer for two-photon PDT [80]. The cage was assembled from six Ru(II)-based photosensitizer ligands and four Pt(II)-based acceptor building blocks, forming a robust supramolecular structure with precise stoichiometry. Owing to the presence of multiple Ru(II) chromophores, the metallacage displayed strong deep-red luminescence and significantly enhanced reactive oxygen species (ROS) generation upon light irradiation, making it particularly suitable for two-photon excitation, which enables deeper tissue penetration and improved spatial control. To further optimize its biological performance, the metallacage was encapsulated within an amphiphilic polymer matrix to form nanoscale particles. This nanoparticle formulation improved aqueous dispersibility, enhanced cellular uptake, and facilitated preferential accumulation in lysosomes, thereby promoting effective intracellular photodynamic action. In vivo experiments conducted in A549 tumor-bearing mice demonstrated pronounced tumor growth inhibition following two-photon irradiation, confirming the therapeutic potential of the metallacage nanoparticles. Notably, the treatment exhibited minimal systemic toxic effects and negligible damage to healthy tissues, highlighting the advantages of combining supramolecular engineering with nanotechnology to develop safer and more effective PDT agents.

**Figure 6:**
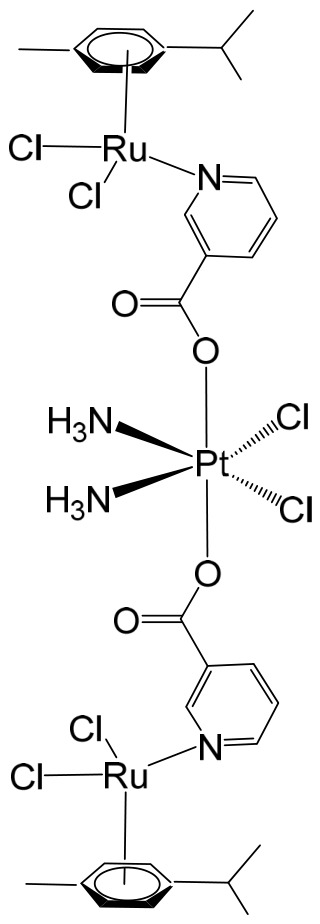
The structure of niacin-bridged platinum(IV)–ruthenium(II) complex.

Building on the strategy of employing Pt(IV) prodrugs to enhance the therapeutic index of more toxic Pt(II) agents, Shu et al. designed and synthesized a series of chimeric complexes incorporating both Pt(IV) and Ru(II) centers connected through a niacin linker, and systematically evaluated their antitumor properties [81]. These niacin-bridged platinum(IV)–ruthenium(II) constructs were rationally assembled from Pt(IV) scaffolds containing a cytotoxic cisplatin core and antimetastatic arene–Ru(II)–chloride fragments. The dual-metal architecture was intended to combine complementary mechanisms of action within a single molecular entity, thereby promoting synergistic anticancer effects while potentially mitigating systemic toxicity. Comprehensive in vitro screening identified the most potent derivative (Figure 6) as a promising lead candidate. This compound exhibited IC_50_ values ranging from 1 to 6.5 μM across multiple human cancer cell lines, including ovarian A2780 cells, indicating strong cytotoxic efficacy. Mechanistic investigations revealed that the water-soluble chimeric prodrug effectively induced apoptosis, predominantly late-stage apoptosis in A2780 cells, and triggered cell-cycle arrest at the G2 phase. Notably, this mechanism differs from that of cisplatin, which is typically associated with S-phase arrest. In addition to its cytotoxic activity, the compound displayed marked antimetastatic and antiangiogenic properties, as demonstrated by significant inhibition in cell invasion and tube-formation assays. These findings suggest that incorporation of the Ru(II) fragment contributes to suppression of metastatic progression and angiogenesis, thereby broadening the therapeutic scope beyond simple tumor growth inhibition.

The in vivo antitumor efficacy of the lead compound (Figure 6) was subsequently assessed in a metastatic A2780 ovarian cancer xenograft model using Balb/c nude mice. Administration at a dose of 3 mg/kg resulted in complete suppression of tumor growth and significantly reduced metastatic dissemination to distant organs. Importantly, its performance surpassed that of the corresponding parent compounds as well as cisplatin under comparable conditions. Beyond its potent inhibition of tumor proliferation and metastasis, the chimeric complex demonstrated a notably improved safety profile in animal studies, exhibiting a higher therapeutic margin than the clinically established platinum drug cisplatin. Collectively, these results highlight the potential of rationally designed Pt(IV)–Ru(II) chimeric systems as multifunctional anticancer agents capable of integrating cytotoxic, antimetastatic, and antiangiogenic activities within a single prodrug framework.

**Figure 7:**
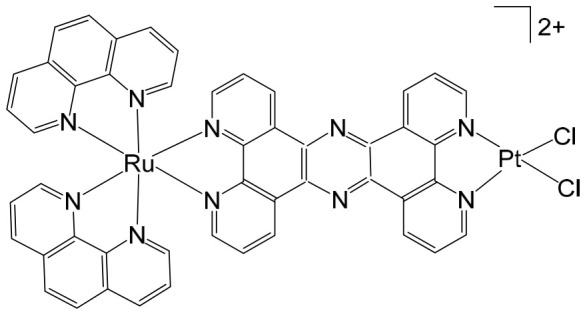
The structure of the tpphz-bridged binuclear heterometallic Ru–Pt complex.

Jarman et al. described a heterobimetallic Ru(II)–Pt(II) complex that acts as a DNA light-switch drug (Figure 7) [82]. They compared the parent cytotoxic complex [Ru(phen)_2_(tpphz)]^2+^ (phen = 1,10-phenanthroline, tpphz = tetrapyridyl [3,2-a:2′,3′-c:3″,2″-h:2‴,3‴-j] phenazine), a mononuclear analog with a modified intercalating ligand [Ru(phen)_2_(taptp)]^2+^, (taptp = 4,5,9,18-tetraazaphenanthreno [9,10-b] triphenylene), and the tpphz-bridged binuclear heterometallic Ru–Pt complex. The study leveraged the distinct DNA interactions of cisplatin versus bis-imine Ru(II) cores, alongside the luminescence enhancement of the Ru complexes upon DNA binding. The heterobimetallic complex displayed lower cytotoxicity than cisplatin in A2780 ovarian cancer cells but showed no significant difference in platinum-resistant A2780cis cells, suggesting that DNA binding is not a primary determinant of platinum resistance. Its optical properties allowed real-time monitoring of DNA interaction dynamics, revealing a switch from intercalation to groove binding and enabling assessment of cell death timing. Live-cell fluorescence microscopy indicated rapid, oncosis-driven cell death, characterized by cytoplasmic volume loss, minimal mitochondrial membrane potential disruption, and absence of apoptotic markers. Quantitative proteomic analysis of A2780 cells treated with the mononuclear complex revealed altered expression of proteins involved in oxidative stress responses and DNA replication/repair pathways. Together, the multi-target activity and induction of non-apoptotic cell death highlight these complexes as promising chemotherapeutic leads.

In various human tumor cell lines, certain ruthenium–platinum heterobimetallic or multimetallic compounds have demonstrated cytotoxic activity that can, in some cases, exceed that of cisplatin, which remains the gold standard among metal-based anticancer drugs. Despite this promising activity, only a small number of these compounds have shown significant antiproliferative effects against cisplatin-resistant tumor cells at sub-micromolar concentrations, highlighting a major challenge in overcoming drug resistance. Furthermore, the often low solubility of these heterometallic compounds restricts their potential for further development as clinically viable antitumor agents.

The exploration of heterobimetallic complexes in cancer therapy is motivated by the desire to combine the distinct properties of different metals within a single molecular framework, thereby potentially enhancing their biological activity and overcoming the limitations of single-metal drugs. One promising approach is the substitution of platinum with other transition metals, which can provide alternative mechanisms of action, improved selectivity, or reduced side effects compared to traditional platinum-based chemotherapy [83,84,85,86,87,88,89,90,91]. By strategically designing these heterometallic systems, researchers aim to create next-generation metal-based therapeutics capable of addressing the challenges of drug resistance and toxicity that continue to limit the clinical utility of conventional platinum compounds.

## 3. Ruthenium–Rhenium Heteronuclear Complexes

Bimetallic metal complexes beyond the well-studied ruthenium–platinum systems have attracted considerable attention in the search for novel therapeutics [92]. One notable strategy involves tethering a reactive PtCl_2_ unit to a mismatch-specific rhenium-centered metallo-intercalator, effectively guiding the cisplatin moiety to selectively react with mismatched DNA sequences [93]. The rhenium precursor itself preferentially targets thermodynamically destabilized mismatched sites and, intriguingly, can induce DNA backbone cleavage upon photoactivation [94]. This approach highlights the potential of combining the complementary properties of different metals to achieve site-specific DNA targeting and controlled reactivity.

In addition to ruthenium, rhenium-based complexes have also emerged as promising antitumor agents due to their unique combination of stability, structural versatility, and low off-target toxicity. These compounds are particularly advantageous in biomedical applications because of their compatibility with real-time imaging techniques, enabling simultaneous therapeutic and diagnostic (“theranostic”) functionality. Although research on Ru(II)–Re(II) heteronuclear complexes has predominantly focused on catalytic applications [95], a limited but growing body of work demonstrates that these complexes can exhibit DNA-switching behavior [82] and pH-responsive luminescence switching [96]. Such properties suggest that heteronuclear complexes could serve as multifunctional platforms, integrating targeted cytotoxicity with real-time monitoring and environmental responsiveness, and thereby broadening the scope of metal-based chemotherapeutics.

**Figure 8:**
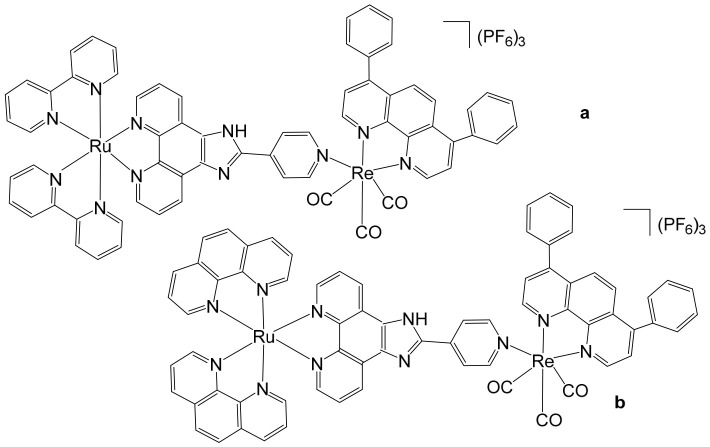
Ru(II)–Re(I) complexes [Ru(bpy)_2_LRe(CO)_3_(DIP)](PF_6_)_3_ (**a**) and [Ru(phen)_2_LRe(CO)_3_(DIP)](PF_6_)_3_ (**b**), where L = 2-(4-pyridinyl) imidazolio [4,5-f][1,10]phenanthroline, bpy = 2,2′-bipyridine, DIP = 4,7-diphenyl-1,10-phenanthroline, phen = 1,10-phenanthroline].

Heterobimetallic complexes incorporating Ru(II) and Re(I) units, specifically [Ru(bpy)_2_LRe(CO)_3_(DIP)](PF_6_)_3_ (Figure 8a) and [Ru(phen)_2_LRe(CO)_3_(DIP)](PF_6_)_3_ (Figure 8b), where L = 2-(4-pyridinyl) imidazolio [4,5-f][1,10]phenanthroline, bpy = 2,2′-bipyridine, DIP = 4,7-diphenyl-1,10-phenanthroline, phen = 1,10-phenanthroline], have been successfully synthesized and systematically evaluated for their anticancer properties by Ma et al. [96]. These heteronuclear Ru(II)–Re(I) complexes exhibited remarkable selectivity toward cancer cells, demonstrating cytotoxic effects superior to that of cisplatin. The observed antiproliferative activity was mediated through multiple interconnected mechanisms, including the induction of apoptosis via cell cycle modulation, mitochondrial membrane depolarization, activation of the caspase cascade, and elevation of intracellular reactive oxygen species (ROS). Detailed mechanistic investigations revealed that these complexes effectively disrupted mitochondrial membrane potential (MMP), leading to mitochondrial damage and the accumulation of ROS within the cell. This oxidative stress further amplified apoptotic signaling, triggering the activation of key caspases and resulting in S-phase cell cycle arrest. Importantly, the enhanced cytotoxicity of these Ru(II)–Re(I) bimetallic complexes was particularly notable in A549R cells, a cisplatin-resistant lung cancer model, highlighting their potential to overcome drug resistance. Beyond inducing apoptosis, these complexes also exerted significant inhibitory effects on cancer cell migration and colony formation, indicating a broader anti-metastatic and anti-proliferative profile. Collectively, these findings underscore the therapeutic promise of Ru(II)–Re(I) heterobimetallic complexes as multifunctional agents capable of targeting both primary tumor growth and metastatic progression.

**Figure 9:**
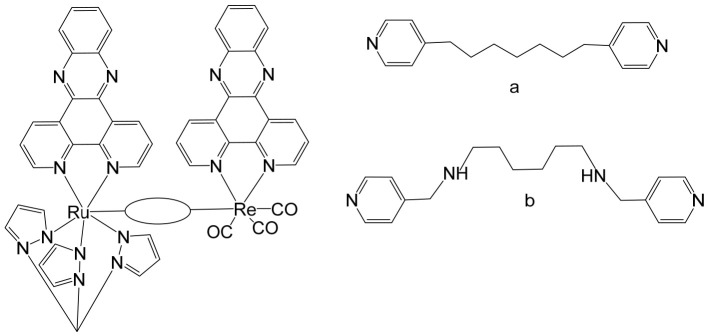
The structures of the complexes [Ru(tpm)dppz-L-Re(CO)_3_dppz]Cl_3_ with a simple dipyridyl alkane (**a**) and N,N’-bis(4-pyridylmethyl)-1,6-hexanediamine (**b**) as linker ligands.

Saeed et al. reported the synthesis and anticancer evaluation of two novel heterobinuclear ruthenium(II)–rhenium(I) complexes [Ru(tpm)dppz-L-Re(CO)_3_dppz]Cl_3_ (Figure 9a,b). These complexes are composed of linked Ru(dppz) and Re(dppz) moieties (dppz = dipyrido [3,2-a:2′,3′-c]phenazine), which are connected via either a simple dipyridyl alkane N,N′-bis(4-pyridylmethyl)-1,6-hexanediamine as the bridging linker ligands [97]. The design of these heterobinuclear systems was intended to combine the photophysical properties of Ru(II) and Re(I) centers, enabling potential synergistic effects in DNA interactions and anticancer activity. Biophysical studies revealed that both complexes bound to DNA with comparable affinities. However, steady-state and time-resolved photophysical measurements indicated that the linker structure strongly modulated the excited state dynamics of the complexes, which in turn influenced their DNA photocleavage efficiency. Computational analyses based on density functional theory (DFT) provided mechanistic insights, showing how linker flexibility and electronic properties affected the photophysical behavior and DNA interaction modes of the complexes. Cellular studies demonstrated that the complexes selectively localized within lysosomes, highlighting a degree of intracellular targeting. Notably, the complex depicted in Figure 9b exhibited enhanced cellular uptake compared to the one in Figure 9a, correlating with superior cytotoxic activity against both A2780 and A2780cisR ovarian cancer cell lines [97]. Furthermore, the complex in Figure 9b showed concentration-dependent localization within the cells, and its distinct photophysical characteristics allowed the intracellular trafficking and localization dynamics to be monitored at super-resolution. These findings underscore the critical role of the linker in tuning not only the photophysical properties but also the biological behavior and anticancer efficacy of heterobinuclear Ru–Re complexes. Overall, this study highlights how careful molecular design of heterobinuclear metal complexes, including the choice of linker, can modulate DNA binding, photocleavage activity, cellular uptake, and subcellular localization, offering a promising strategy for the development of targeted phototherapeutic agents.

The coordination bond–directed self-assembly approach enables the construction of structurally robust and photoactive Ru(II)-based architectures (heteronuclear square metallocycles), including rhenium, that demonstrate promising DNA-binding capabilities and potent anticancer activity. These unique metallocycles offer a versatile platform for exploring molecular recognition, photochemical reactivity, and targeted therapeutics. However, their broader applicability is severely constrained by low synthetic yields and the need for labor-intensive, costly purification procedures, underscoring the urgent requirement for more efficient and scalable synthetic methodologies.

**Figure 10:**
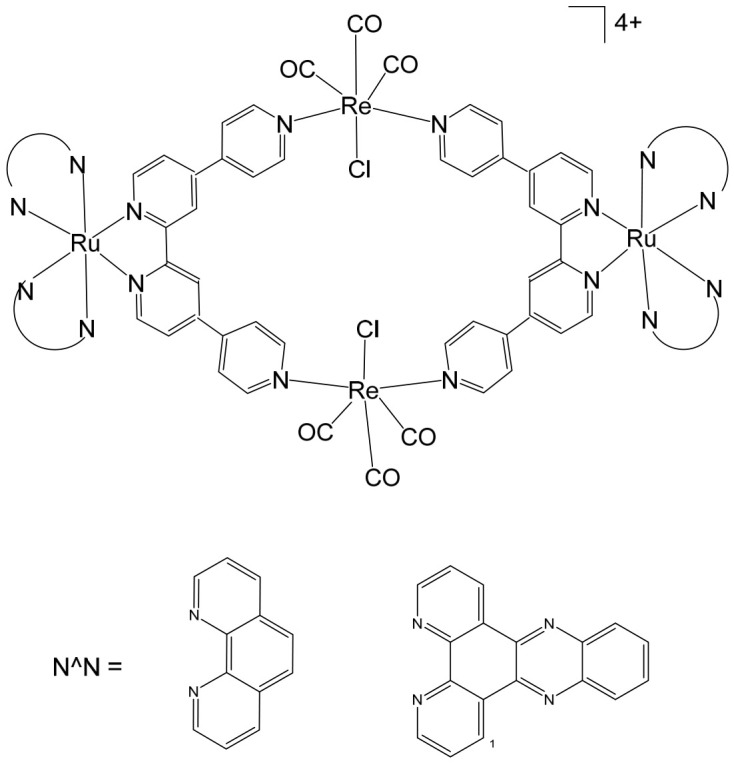
The structure of tetranuclear metallomacrocycles with 2,2′:4,4″:4′,4‴-quaterpyridyl (qtpy) bridging ligand.

The synthesis [98] and detailed structural characterization [99] of self-assembled, kinetically inert, water-soluble ruthenium–rhenium metallomacrocycles, which are based on low-spin d^6^ metal ions such as ruthenium(II) and rhenium(I), have been extensively reported. These metallomacrocycles exhibit remarkable stability in aqueous environments, maintaining their integrity under biologically relevant conditions. Their architectures feature highly organized and well-defined binding pockets formed by hydrophobic aromatic residues, which provide selective and high-affinity interactions with a variety of biomolecules [100]. Among these, kinetically inert tetranuclear metallomacrocycles (Figure 10), constructed using the 2,2′:4,4″:4′,4‴-quaterpyridyl (qtpy) bridging ligand, have been investigated in detail by Ahmad et al. [101]. These complexes display a strong ability to bind duplex DNA, employing a non-intercalative external binding mode that is reminiscent of natural DNA recognition proteins, such as the TATA box binding protein. This interaction not only allows high-affinity binding but also induces significant conformational changes in the DNA, including large-scale bending, highlighting the potential of these metallomacrocycles in biomolecular recognition and DNA-targeted applications [101].

**Figure 11:**
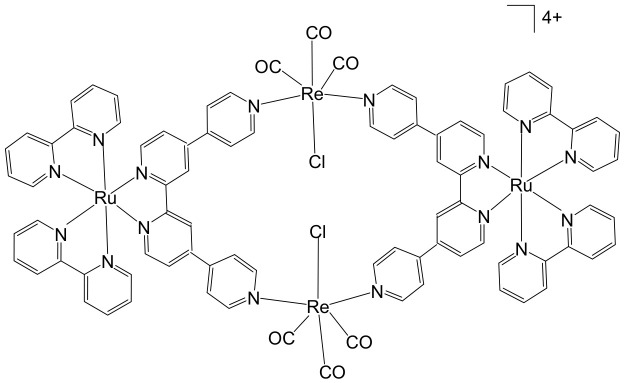
Modified tetranuclear metallomacrocycles.

The same authors explored the previously reported macrocycles (Figure 10) alongside the heterometallic complex depicted in Figure 11 to investigate how modifications in the ancillary ligand environment surrounding the central macrocyclic core influence DNA-binding properties. Both classes of macrocycles (Figure 10 and Figure 11) demonstrated notable affinities for anions and aromatic systems, highlighting their potential as versatile molecular hosts. These shared binding characteristics suggested that the complexes were particularly well-suited for studying how extended aromatic ancillary ligands might modulate both the strength and mode of interaction with nucleic acids. To examine these effects, water-soluble chloride salts of the macrocycles, shown in Figure 10, were synthesized. Their interactions with duplex DNA in aqueous buffer solutions were systematically compared with those of the heterometallic complex in Figure 11 [100]. This comparison aimed not only to quantify DNA affinity but also to assess whether modifications in the ligand framework could influence binding selectivity or induce distinct structural interactions with the nucleic acid. In parallel, Ahmad et al. have described the self-assembly of a “kinetically locked” luminescent heterometallic macrocycle with luminescent properties (Figure 11) that functions as a host for polyaromatic molecules, anions, and biopolymers [101]. Detailed structural studies revealed a large, highly organized binding cavity composed predominantly of hydrophobic aromatic residues, capable of stabilizing a variety of guest molecules through π–π interactions and electrostatic complementarity. Given its pronounced affinity for both aromatic systems and anionic species, the interaction of this macrocycle with nucleotide anions was investigated in detail. These studies demonstrated that the macrocycles could act as sensitive luminescent sensors for nucleotide triphosphates. Notably, one of the hosts exhibited a distinctive “off–on” luminescent response upon ATP binding, indicating a selective recognition mechanism. Such behavior underscores the potential of these heterometallic macrocycles not only as DNA-binding agents but also as functional probes for biologically relevant anions, bridging supramolecular chemistry and bioanalytical applications.

Given that these macrocycles integrate polypyridyl Ru(II) and Re(I) units closely related to systems currently being explored as photosensitizers for photodynamic therapy (PDT), the authors conducted a broader evaluation of their biological behavior, including cellular uptake and photocytotoxic activity against human cancer cell lines. Under clinically relevant irradiation conditions, the Ru(II)–Re(I) metallomacrocycles demonstrated efficient cellular internalization and pronounced photocytotoxicity. Their activity was attributed to the light-triggered generation of reactive oxygen species (ROS), which induced significant plasma membrane damage and ultimately led to cancer cell death [100,101]. In 2016, Walker and co-workers reported that a Ru(II)–Re(I) metallomacrocycle (Figure 11) acts as an efficient intracellular singlet oxygen (^1^O_2_) sensitizer for photodynamic therapy (PDT) [102]. Given that this class of macrocycles integrates polypyridyl Ru(II) and Re(I) centers closely related to established and emerging PDT sensitizers, the authors conducted a comprehensive investigation of its cellular uptake, subcellular localization, DNA-binding affinity, and photocytotoxic activity in human tumor cell lines. Under clinically relevant light irradiation conditions, the Ru(II)–Re(I) metallomacrocycle was readily internalized by cancer cells and functioned as a highly effective photosensitizer. Upon light activation, it generated reactive oxygen species (ROS), leading to significant plasma membrane damage and subsequent cell death. In addition to its photochemical activity, the metallomacrocycle (Figure 11) demonstrated strong interactions with biological targets, exhibiting very high DNA-binding affinity (>10^6^ M^−1^) [103], which may further contribute to its biological effects. Compared with the corresponding mononuclear Ru(II) module, the metallomacrocycle displayed markedly enhanced intracellular accumulation, approximately sevenfold higher. Fluorescence imaging revealed intense staining in lipid-rich regions, including the nuclear membrane and endoplasmic reticulum, indicating preferential localization in membrane-associated environments. This distinct uptake and localization behavior sharply contrasted with that of the mononuclear analog. Both the metallomacrocycle (Figure 11) and the mononuclear Ru(II) complex exhibited minimal dark toxicity, highlighting their relative safety in the absence of light. However, upon photoactivation, only the metallomacrocycle showed pronounced cytotoxicity against the cisplatin-resistant ovarian cancer A2780cisR cell line, with an IC_50_ value of approximately 0.3 μM. Notably, this value is nearly two orders of magnitude lower than that of cisplatin (IC_50_ = 22.9 μM) and the mononuclear Ru(II) module (IC_50_ = 22.9 μM), underscoring its superior phototherapeutic efficacy. The substantial difference in photocytotoxic performance was primarily attributed to the enhanced cellular uptake and favorable subcellular distribution of the metallomacrocycle relative to its mononuclear counterpart.

## 4. Ruthenium–Gold Heteronuclear Complexes

Systems incorporating Ru and Au centers bridged by a variety of ligands have attracted considerable attention in recent years, and numerous studies have demonstrated that these bimetallic assemblies frequently display superior biological activity compared to their monometallic counterparts [104,105,106]. The enhanced performance of such systems is often attributed to synergistic effects arising from the cooperative interaction between the two metal centers, which can modulate electronic properties, redox behavior, stability, and cellular uptake. Gold complexes, in particular, represent a highly promising class of metallodrugs for cancer chemotherapy. Unlike platinum-based drugs, gold compounds are not primarily DNA-targeting agents. Instead, gold has been identified as an efficient cytotoxic center whose mechanism of action is believed to involve disruption of mitochondrial function, induction of oxidative stress through the generation of reactive oxygen species (ROS), and inhibition of thiol-containing enzymes. This distinct mode of action allows gold-based compounds to circumvent classical cisplatin resistance mechanisms in certain cancer cell lines [104]. To further potentiate anticancer efficacy, several research efforts have focused on the rational design of heterometallic systems in which Ru and Au centers are covalently linked through diphosphine or related bridging ligands [105,106]. Such architectures are designed to integrate the complementary biological profiles of both metals: the versatile coordination chemistry and redox activity of ruthenium, together with the strong affinity of gold for sulfur- and selenium-containing biomolecules. By combining these properties within a single molecular framework, Ru–Au complexes offer the potential for dual or multi-target mechanisms, improved selectivity, and enhanced therapeutic performance relative to individual monometallic species.

**Figure 12:**
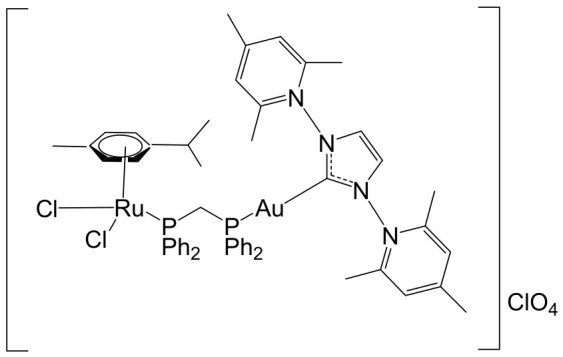
The structure of heterometallic ruthenium–gold complex [Cl_2_(p-cymene)Ru(μ-dppm)Au(IMes)]ClO_4_.

The ruthenium–gold class of heterometallic complexes incorporating Au–N-heterocyclic carbene (NHC) fragments represents an important strategy in the development of multifunctional anticancer agents. These systems, exemplified by the general formula [Cl_2_(p-cymene)Ru(μ-dppm)Au(NHC)]ClO_4_, are designed to combine the complementary pharmacological properties of both metal centers within a single molecular framework. Such dual-metal architectures have been reported to exhibit synergistic biological effects that surpass those of the corresponding monometallic counterparts. Biological investigations have demonstrated that these Ru–Au–NHC complexes display pronounced cytotoxic and antiproliferative activity across a range of cancer cell lines. In addition to direct growth inhibition, they exhibit marked antimetastatic, antimigratory, and antiangiogenic properties, highlighting their potential to interfere not only with tumor cell viability but also with processes associated with cancer progression and dissemination. In vitro studies have shown that specific representatives of this class, particularly [Cl_2_(p-cymene)Ru(μ-dppm)Au(IMes)]ClO_4_, possess significant chemotherapeutic potential against colorectal and renal carcinoma cell lines. Elie et al. reported the synthesis and detailed characterization of this heterometallic ruthenium–gold complex [Cl_2_(p-cymene)Ru(μ-dppm)Au(IMes)]ClO_4_ (Figure 12), where IMes corresponds to 1,3-bis(2,4,6-trimethylphenyl)imidazol-2-ylidene [107]. Structural and spectroscopic analyses confirmed the successful incorporation of both the Ru(II) arene fragment and the Au(I)–NHC moiety within the same coordination framework. The conceptual design of these anticancer gold–ruthenium heterometallic systems is based on merging the well-documented antimetastatic and cytotoxic properties of the [(p-cymene)RuCl_2_(μ-dppm)] fragment with the antimitochondrial activity and thioredoxin reductase-inhibiting effects associated with cationic [Au(NHC)]^+^ and [Au(NHC)(PR_3_)]^+^ species [108]. By integrating these complementary mechanisms of action, Ru–Au complexes aim to target multiple intracellular pathways simultaneously, thereby enhancing therapeutic efficacy and potentially overcoming resistance mechanisms. Moreover, ruthenium–gold compounds have been evaluated in comparison with clinically relevant gold-based drugs such as Auranofin [109], further underscoring their promise as next-generation metal-based chemotherapeutic agents.

The studies were conducted in vitro using the human clear cell renal carcinoma (ccRCC) cell line Caki-1. The findings clearly demonstrated that the Ru–Au bimetallic compound (Figure 12) exhibited significantly greater cytotoxicity than its corresponding monometallic ruthenium or gold analogs, highlighting the therapeutic advantage of the heterometallic strategy. Beyond its enhanced cytotoxic profile, the compound effectively inhibited key processes required for metastatic progression, including cellular migration, invasion, and angiogenesis. Mechanistically, the Ru–Au complex disrupted pericellular proteolysis by inhibiting several proteolytic enzymes critically involved in extracellular matrix remodeling and tumor dissemination. These included cathepsins and metalloproteinases, such as matrix metalloproteinases (MMPs) and a disintegrin and metalloproteinases (ADAMs), both of which play central roles in cancer pathogenesis, tumor invasion, and metastatic niche formation. In addition, the compound inhibited the mitochondrial enzyme thioredoxin reductase (TrxR), a selenoprotein frequently overexpressed in cancer cells and associated with redox homeostasis, proliferation, and resistance to apoptosis. Inhibition of TrxR contributed to increased oxidative stress and subsequent induction of apoptotic cell death. Although auranofin produced comparable effects on migration and invasion in Caki-1 cells, the Ru–Au compound displayed a broader and more distinctive biological profile. Notably, it uniquely suppressed angiogenic tube formation and significantly reduced vascular endothelial growth factor (VEGF) expression, indicating a stronger antiangiogenic capacity. Furthermore, comparative analyses revealed that auranofin and the Ru–Au complex generated distinct proteolytic modulation patterns, suggesting differences in their molecular targets and downstream signaling pathways. Collectively, these findings position the Ru–Au complex as a highly promising candidate for further preclinical development in renal cancer therapy. The heterometallic Ru–Au compound (Figure 12) was rationally engineered to integrate complementary pharmacological properties within a single molecular framework. Specifically, it combines the cytotoxic and pro-apoptotic effects of Au(I) lipophilic cations, including potent TrxR inhibition, with the anticipated antimetastatic and signaling-modulatory properties of a Ru p-cymene phosphine fragment. This design aimed to exploit synergistic interactions between the two metal centers to enhance therapeutic efficacy while broadening the spectrum of molecular targets. In functional assays, the compound produced substantial inhibition of migration (82%), invasion (66%), and angiogenesis, demonstrating robust suppression of metastatic behavior. At the molecular level, it modulated multiple signaling mediators implicated in tumor progression, including various interleukins, metalloproteinases, and cathepsins associated with metastasis and angiogenesis. Particularly strong inhibition was observed for VEGF, a central driver of neovascularization and tumor growth. Importantly, the overall biological activity of the heterometallic complex generally surpassed that of the individual monometallic fragments. In certain cases, specific effects could be attributed predominantly to one metal center, further underscoring the synergistic and cooperative contribution of the dual-metal architecture. The in vivo antitumor efficacy of the Ru–Au compound was evaluated in NOD.CB17-Prkdc SCID/J mice bearing 21-day xenograft ccRCC Caki-1 tumors. Treatment with the compound at a dose of 10 mg/kg administered every 72 h resulted in complete tumor growth inhibition, with no observable pathological complications or signs of systemic toxicity [110]. These findings indicate a favorable therapeutic index and support further translational investigation. At the molecular level in vivo, treatment led to a significant downregulation of growth factors associated with malignant progression and aggressive tumor phenotypes. These included VEGF (angiogenesis), platelet-derived growth factor (PDGF), fibroblast growth factor (FGF), epidermal growth factor receptor (EGFR), and hepatocyte growth factor receptor (HGFR), all of which contribute to tumor cell proliferation, survival signaling, and protumorigenic activity. The coordinated suppression of these pathways reinforces the compound’s multitargeted mechanism of action. Overall, the heterobimetallic Ru–Au complex (Figure 12) demonstrates a broad-spectrum antitumor profile encompassing cytotoxic, pro-apoptotic, antiangiogenic, and antimetastatic activities. Its potent inhibition of key proteolytic enzymes, such as MMPs, ADAMs, and cathepsins, further supports its potential to limit metastatic dissemination, consistent with previous reports linking the inhibition of these factors to reduced metastasis [110]. Taken together, the preclinical data strongly support continued development of this heterometallic platform as a promising therapeutic strategy for advanced renal cancer.

**Figure 13:**
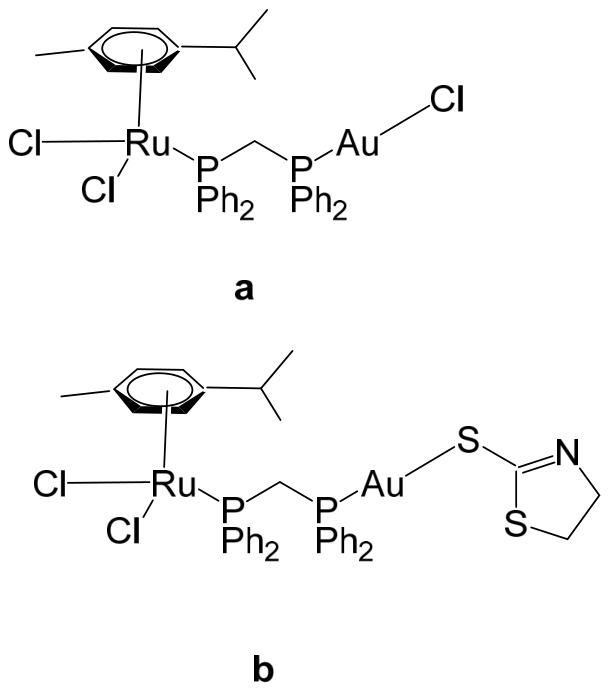
Structures of Ru(II)-Au(I) complexes [RuCl_2_(p-cymene)(µ-dppm)AuCl] (**a**) and [RuCl_2_(p-cymene)(µ-dppm)Au(S-thiazoline)] (**b**).

Massai et al. have reported the preparation, characterization and stability assessment of a series of bimetallic Ru(II)–Au(I) complexes. These compounds were designed by combining two cytotoxic metal fragments: the ruthenium(II) p-cymene dichloride moiety, [Ru(p-cymene)Cl_2_(PR_3_)], known for its antimetastatic and antiproliferative properties, and gold(I) chloride or thiolate scaffolds, [AuX(PR_3_)] (X = Cl, SR), which are also recognized for their cytotoxic potential. The two metal centers were linked through the bifunctional diphosphane ligand 1,1-bis(diphenylphosphino) methane (dppm), allowing precise spatial organization of the heterometallic system [111]. The biological activity of two representative heterobimetallic complexes, [RuCl_2_(p-cymene)(µ-dppm)AuCl] (Figure 13a) and [RuCl_2_(p-cymene)(µ-dppm)Au(S-thiazoline)] (Figure 13b), was evaluated against the human colon carcinoma HCT116 cell line, a model of aggressive colorectal cancer, as well as the non-cancerous L929 mouse fibroblast cell line. These results were directly compared to the corresponding mononuclear ruthenium and gold species to assess the potential enhancement provided by heterobimetallic assembly. Remarkably, the heterobimetallic compounds demonstrated superior in vitro pharmacological profiles relative to their monometallic counterparts [Ru(p-cymene)Cl(µ-Cl)]_2_ and [Ru(p-cym)Cl_2_ (η^1^-dppm)]. They exhibited higher cytotoxicity or selectivity toward cancer cells, highlighting the potential of bimetallic synergy. The cytotoxic effects were strongly dose-dependent, indicating precise tunability of their pharmacological behavior. Importantly, the non-cancerous L929 fibroblasts showed significantly lower sensitivity to these compounds, suggesting a favorable therapeutic window. To further explore their mechanism of action, preliminary studies were conducted to assess interactions with common biomolecular targets, including DNA, the lysosomal enzyme cathepsin B, and selected model proteins. These investigations provide early insights into the multifaceted ways in which these heterobimetallic complexes may exert their antiproliferative and potentially antimetastatic effects, supporting their promise as next-generation chemotherapeutic candidates.

**Figure 14:**
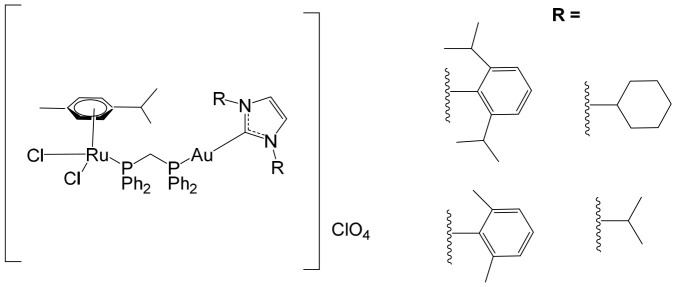
Ruthenium(II)–gold(I) cationic complexes of the type [(η^6^-p-cym)RuCl_2_(μ-dppm)Au(NHC)]ClO_4_ incorporating different gold(I)–N-heterocyclic carbene ligands (NHC).

To further improve the pharmacological profile of the Ru(II)–Au(I) complexes shown in Figure 13, the same authors expanded their research to the efficient synthesis and biological evaluation of a broader family of ruthenium(II)–gold(I) cationic complexes with the general formula [(η^6^-p-cym)RuCl_2_(μ-dppm)Au(NHC)]ClO_4_ (Figure 14). These heterobimetallic systems incorporate a range of structurally diverse gold(I) N-heterocyclic carbene (NHC) ligands into the [Ru(p-cymene)Cl_2_ (η^1^-dppm)] framework [112], thereby widening the structural and electronic variability of the complexes. This design strategy is based on the rationale of integrating complementary biological properties within a single molecular platform. In particular, the potential antimetastatic and cytotoxic activity associated with [Ru(p-cymene)Cl_2_ (η^1^-dppm)] is combined with the well-documented antimitochondrial effects and thioredoxin reductase (TrRx) inhibitory activity of cationic gold(I) species such as [(NHC)Au]^+^ and [(NHC)Au(PR_3_)]^+^. By merging these distinct yet synergistic mechanisms of action, the resulting Ru(II)–Au(I) conjugates represent promising candidates for the development of more effective and multifunctional cancer chemotherapeutic agents.

These compounds exhibited remarkable stability under physiological conditions and pronounced cytotoxic activity against renal Caki-1 and colon HCT116 and DLD-1 cancer cell lines. Importantly, they displayed clear cytotoxic selectivity when compared with the non-tumorigenic kidney HEK 293T cell line. Collectively, these results indicate that Au complexes preferentially target malignant cells while exerting minimal effects on healthy counterparts, in agreement with multiple previous reports [113]. Preliminary mechanistic investigations further revealed that these compounds operate via a mode of action distinct from that of cisplatin. Rather than interacting with DNA, they behave more similarly to other Au(I) derivatives bearing lipophilic ligands, such as phosphane-containing compounds (e.g., Auranofin) and N-heterocyclic carbene complexes, as described elsewhere [114]. The compounds (Figure 14) did not exhibit measurable DNA binding but instead selectively inhibited mitochondrial thioredoxin reductase in Caki-1 renal cancer cells, highlighting a mitochondria-centered mechanism of cytotoxic action.

**Figure 15:**
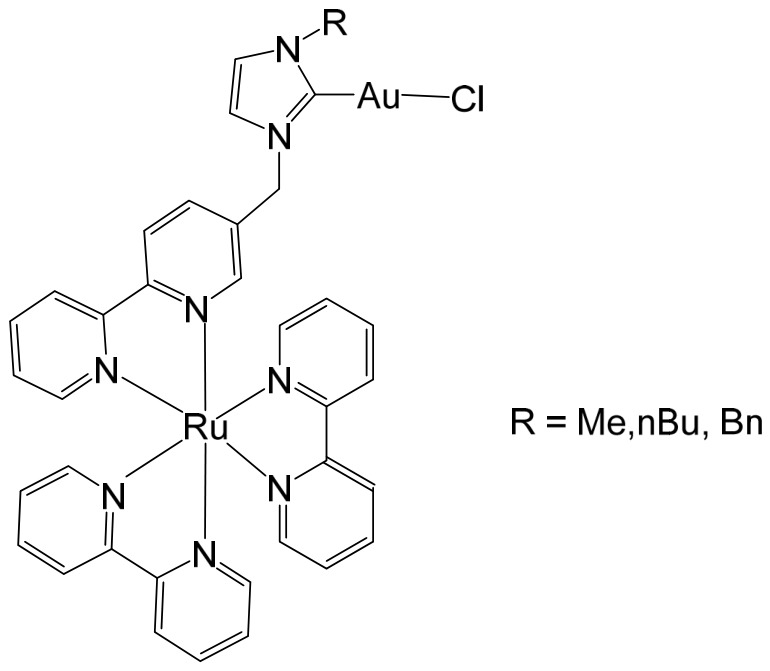
Heterobimetallic gold(I)–ruthenium(II) complexes containing different heteroditopic bipyridine–N-heterocyclic carbene (NHC) ligands, where R = Me (a), nBu (b) and Bn (c).

Boselli et al. reported the synthesis and characterization of heterometallic complexes featuring heteroditopic bipyridine–N-heterocyclic carbene (NHC) ligands, designed to combine the photophysical properties of an emissive Ru(II) center with the biological activity of a Au(I) fragment [115]. Their strategy involved functionalization of the well-known luminescent [Ru(bipy)_2_(N^N)]^2+^ scaffold (where N^N denotes a diimine ligand) with an imidazole derivative. This modification enabled subsequent coordination of the Au(I) unit through formation of the NHC, thereby generating a structurally integrated Ru–Au system (Figure 15). Given the strong cytotoxic profiles commonly associated with Au–NHC complexes [116], the resulting heterobimetallic species were anticipated to display enhanced anticancer activity. However, although complexes depicted in Figure 15 exhibited measurable bioactivity, their cytotoxic performance did not exceed that of the clinically approved multikinase inhibitor Sorafenib, which was used as a benchmark. These findings suggest that the incorporation of the Au(I) fragment did not produce the expected synergistic enhancement in therapeutic efficacy. Despite the moderate cytotoxic outcomes, the photophysical investigations provided particularly noteworthy results. Importantly, coordination of the Au(I) fragment did not quench the characteristic Ru(II)-centered emission. On the contrary, the presence of Au(I) led to an increase in the emission quantum yield, indicating a beneficial electronic interaction between the two metal centers. This enhancement preserves, and even improves, the luminescent properties of the Ru(II) core, underscoring the potential of these complexes as theranostic agents that combine imaging capability with therapeutic function. Cellular localization studies further supported their bioimaging applicability. In experiments performed on Hep3B liver cancer cells, complex (Figure 15) was observed to accumulate predominantly in the cytoplasm, especially in the perinuclear region. This subcellular distribution pattern may provide valuable insights into its mechanism of action and highlights the suitability of these heterometallic systems for intracellular tracking and visualization studies.

**Figure 16:**
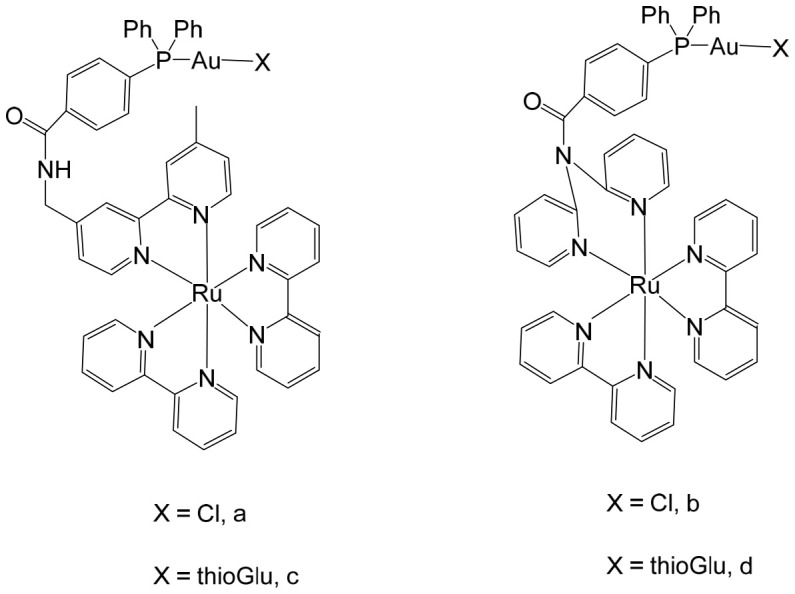
Heterodinuclear gold(I)–ruthenium(II) luminescent complexes with different organic ligands.

Subsequently, closely related heterodinuclear luminescent systems incorporating alternative organic linkers were described by Wenzel et al. [117]. These architectures integrate a luminescent Ru(II) polypyridyl chromophore with a metal-based anticancer fragment; namely, AuCl, Au-thioglucose and (*p*-cymene)RuCl_2_ (Figure 16). A key design strategy consisted of tailoring the gold coordination environment to closely mimic that of Auranofin, thereby aiming to enhance therapeutic performance. To this end, the bifunctional diimine ligand bridging the two metallic centers was equipped with a phosphine donor site capable of binding the Au(I) ion, reproducing structural features associated with biologically active gold compounds. In parallel, the photophysical behavior of the Ru(II) fragment was fine-tuned by replacing the previously employed bipyridine ligand with 2,2′-dipyridylamine. This modification was intended to modulate both the electronic properties and emission characteristics of the chromophore, potentially improving imaging capabilities while maintaining biological activity.

The full series of bimetallic complexes was assessed in vitro for antiproliferative activity against human cancer cell lines. A clear structure–activity relationship emerged: only derivatives incorporating the Au(I) fragment displayed significant cytotoxic effects, with activities comparable to or exceeding those of cisplatin. In particular, substitution of chloride by thioglucose (Figure 16) led to a marked increase in cytotoxicity relative to the chloride-containing analogs (Figure 16). These findings underscore the decisive role played by the ancillary ligands coordinated to gold in dictating biological efficacy. Comprehensive photophysical and electrochemical investigations revealed that all complexes were emissive in solution. Among them, the species shown in Figure 16 exhibited the highest emission intensities, highlighting the influence of ligand environment on excited-state properties. Importantly, cellular uptake studies demonstrated that these bimetallic constructs permeate cancer cells through an active transport mechanism rather than passive diffusion. The most promising compounds, those featuring the Au–thioglucose scaffold, were efficiently internalized at physiological temperature (37 °C) and localized within the cytoplasm or specific subcellular compartments. Notably, the two thioglucose derivatives displayed distinct intracellular distributions: the complex depicted in Figure 16 accumulated predominantly in the cytoplasm, whereas the analog in Figure 16 was found within the nucleus and associated organelles. This differential localization indicates that cellular biodistribution is governed not solely by the bioactive Au(I) fragment, but also by the structural and physicochemical characteristics of the luminescent Ru(II) core, emphasizing the cooperative contribution of both metal centers to the overall biological profile.

## 5. Ruthenium–Iron Complexes

The anticancer properties of ruthenium complexes have long been associated with their presumed interaction with transferrin, the principal iron transport protein in blood plasma. This hypothesis stems from the chemical similarities between ruthenium and iron, particularly their comparable coordination chemistry, which suggested that ruthenium compounds might exploit endogenous iron transport pathways to reach tumor tissues. However, accumulating experimental evidence indicates that, upon entering the bloodstream, ruthenium complexes rapidly and preferentially bind to human serum albumin (HSA), the most abundant plasma protein. Given that HSA is present at concentrations far exceeding those of transferrin, this interaction substantially restricts the fraction of ruthenium complexes available for transferrin-mediated transport. Consequently, the original assumption of transferrin as the primary carrier may represent an oversimplification of the in vivo behavior of these compounds. Despite this limitation, the strategic targeting of iron metabolism remains a compelling approach in anticancer drug design. Iron plays a pivotal role in oncogenesis, supporting DNA synthesis, cellular respiration, and the rapid proliferation that characterizes malignant cells. Cancer cells frequently exhibit dysregulated iron homeostasis and enhanced iron uptake to sustain their metabolic demands. These alterations create vulnerabilities that can be therapeutically exploited. In particular, the elevated expression of transferrin receptors and increased iron acquisition mechanisms in tumors may provide selective entry pathways for metal-based therapeutics, thereby enhancing tumor specificity while limiting systemic toxicity. Within this broader context, iron-containing and iron-mimicking agents have gained renewed attention as potential antineoplastic compounds. Of particular interest are Fe–Ru bimetallic complexes, which integrate the distinct yet complementary properties of both metals within a single molecular framework. By combining the redox activity and biological relevance of iron with the unique coordination chemistry and cytotoxic potential of ruthenium, these hybrid systems may offer synergistic mechanisms of action. Emerging studies suggest that such bimetallic complexes constitute a promising new class of anticancer agents, with the potential to overcome some of the pharmacokinetic and mechanistic limitations associated with monometallic ruthenium compounds.

**Figure 17:**
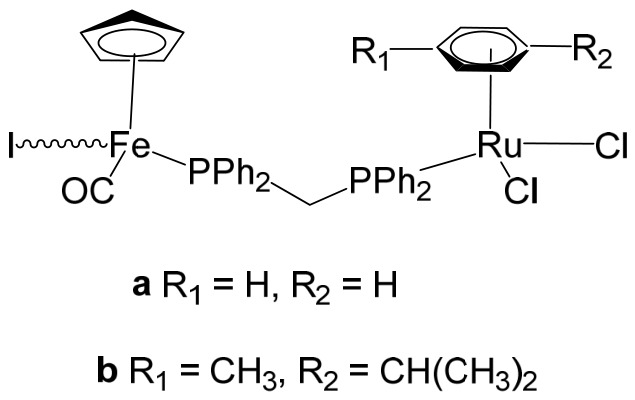
Structures of heterobimetallic μ-dppm bridged Fe–Ru complexes [(η^6^-C_6_H_6_)RuCl_2_(μ-dppm)Fe(CO)I(η^5^-C_5_H_5_)] (a) and [(η^6^-p-cym)RuCl_2_(μ-dppm)Fe(CO)I(η^5^-C_5_H_5_)] (b).

Herry et al. have reported a series of heterobimetallic μ-dppm bridged Fe–Ru complexes with the general formulas [(η^6^-C_6_H_6_)RuCl_2_(μ-dppm)Fe(CO)I(η^5^-C_5_H_5_)] and [(η^6^-p-cym)RuCl_2_(μ-dppm)Fe(CO)I(η^5^-C_5_H_5_)] (Figure 17) [91]. In these complexes, the bridging ligand dppm (1,1-bis(diphenylphosphino)methane) connects a ruthenium center coordinated to an η^6^-arene and two chlorides to an iron center bound to a cyclopentadienyl ring, a carbonyl, and an iodide. Specifically, the complexes [(η^6^-arene)RuCl_2_(μ-dppm)Fe(CO)I(η^5^-C_5_H_5_)] (Ar = C_6_H_6_ (Figure 17a) and [(η^6^-p-cymene)RuCl_2_(μ-dppm)Fe(CO)I(η^5^-C_5_H_5_)] (Figure 17b) were synthesized through the reaction of [Fe(η^5^-C_5_H_5_)I(CO)(k^1^-dppm)] with the corresponding dimeric ruthenium precursors [(η^6^-Arene)RuCl_2_]_2_.

The structural and electronic properties of these heterobimetallic complexes were thoroughly characterized using a combination of spectroscopic techniques, including NMR and IR spectroscopy, and density functional theory (DFT) calculations. These analyses confirmed the expected coordination geometries around both metal centers and provided insight into electronic interactions between the Fe and Ru centers mediated by the μ-dppm bridge. To assess their potential biological activity, the complexes were tested for cytotoxicity against human ovarian carcinoma cell lines A2780 (cisplatin-sensitive) and A2780cisR (cisplatin-resistant), as well as non-tumorigenic human embryonic kidney HEK293 cells. Their cytotoxic effects were compared not only to the clinically used chemotherapeutic cisplatin, but also to related organometallic complexes, including the homodinuclear Ru–Ru complex [(η^6^-C_6_H_6_) RuCl_2_(μ-dppm)RuCl_2_(η^6^-C_6_H_6_)] and the mononuclear analogs [(η^6^-C_6_H_6_)RuCl_2_(k^1^-dppm)] and [Fe(η^5^-C_5_H_5_)I(CO)(k^1^-dppm)]. Both complexes, shown in Figure 17a,b, demonstrated remarkably high cytotoxic activity in both A2780 and A2780cisR cells, outperforming cisplatin in both cell lines. Notably, the complex (Figure 17b) was slightly more potent than the complex (Figure 17a), showing IC_50_ values of 1.4 μM versus 2.2 μM in A2780 cells and 1.2 μM versus 1.5 μM in A2780cisR cells. These results indicate that substitution of the arene ligand from benzene to p-cymene can modestly enhance biological activity, potentially through altered cellular uptake or changes in lipophilicity. The enhanced cytotoxicity of these heterobimetallic Fe–Ru complexes relative to homodinuclear Ru–Ru analogs suggests a synergistic effect arising from the combination of two different metal centers. Although the precise mechanism of action is not yet fully understood, the authors proposed that the presence of the iron center may facilitate greater cellular uptake in malignant cells, possibly through ferritin-mediated transport pathways, thereby contributing to the higher activity observed. These findings highlight the potential of heterobimetallic organometallic complexes as a promising strategy for designing next-generation anticancer agents that could overcome resistance to conventional chemotherapeutics.

**Figure 18:**
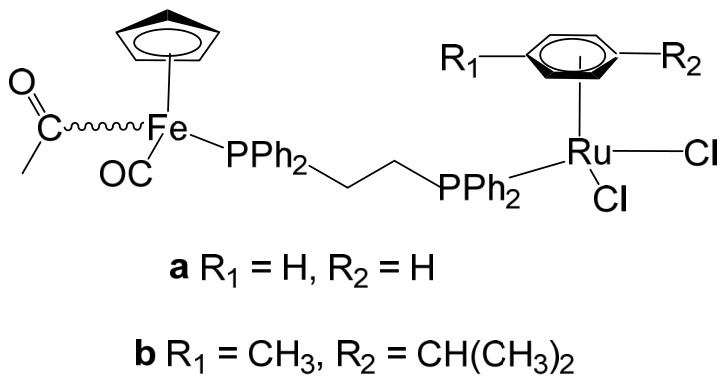
Structures of heterobimetallic Fe–Ru complexes [CpFe(CO)(COCH_3_)(μ-dppe)Ru(η^6^-C_6_H_6_)Cl_2_] (a) and [CpFe(CO)(COCH_3_)(μ-dppe)Ru(η^6^-p-cym)Cl_2_] (b).

A later study explored how modifications to the molecular structure, specifically increasing the spacer length between the metal centers and introducing greater lipophilicity through the substitution of iodine (I) with an acetyl group (COCH_3_), affected the properties of heterobimetallic complexes. In this context, the Fe–Ru complexes [CpFe(CO)(COCH_3_)(μ-dppe)Ru(η^6^-C_6_H_6_)Cl_2_] (Figure 18a) and [CpFe(CO)(COCH_3_)(μ-dppe)Ru(η^6^-p-cym)Cl_2_] (Figure 18b) were synthesized. Both compounds incorporated the 1,1-bis(diphenylphosphino)ethane (dppe) ligand, which is known to influence both the geometry and electronic properties of the metal centers [118]. The cytotoxicity of these complexes was assessed against the cisplatin-resistant ovarian carcinoma cell line A2780cisR. The results mirrored earlier observations made with related dppm-containing complexes (Figure 17a,b), in which subtle changes in the ligand environment and the nature of the arene affected biological activity. Specifically, the complex in Figure 18b, which features a p-cymene ligand, displayed higher cytotoxicity than its benzene analog (Figure 18a), with IC_50_ values of 4.9 μM and 6.5 μM, respectively. This suggests that the substitution of the arene and the resulting changes in steric and electronic properties can significantly influence the interaction of these complexes with cancer cells. Interestingly, both heterobimetallic Fe–Ru complexes outperformed cisplatin in this model and were markedly more active than Fe–Fe analogs, which exhibited very low cytotoxicity. A broader comparison of dppe-based complexes (Figure 18) with their dppm-based counterparts (Figure 17) revealed a decline in cytotoxicity upon switching to dppe. This decrease may be attributed to one or a combination of structural factors: the longer spacer in dppe potentially reduces the effective interaction with cellular targets, while the replacement of iodine with the COCH_3_ group alters the overall lipophilicity and electronic distribution in the complex, thereby influencing cellular uptake and activity. These findings underscore the delicate balance between ligand structure, metal coordination, and cytotoxic efficacy, highlighting how even minor modifications in heterobimetallic complexes can have pronounced effects on biological activity.

**Figure 19:**
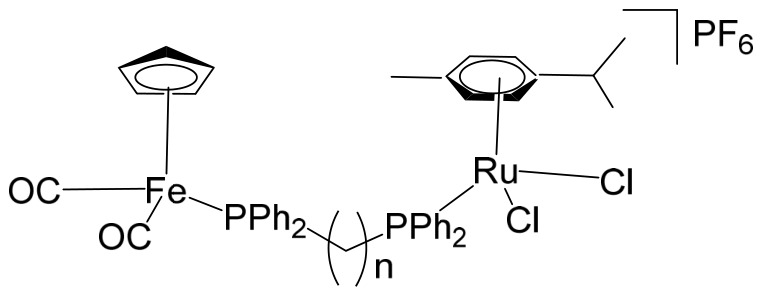
The structure of heterobimetallic Fe–Ru salt complexes of diphosphine ligands of varying chain lengths, where n = 2 (a), n = 3 (b), n = 4 (c), n = 5 (d).

Odachowski et al. have investigated heterobimetallic Fe–Ru salt complexes in which the metal centers are bridged by diphosphine ligands of varying chain lengths [119]. Their previous work had explored the influence of using neutral versus salt complexes on anticancer activity. These studies revealed that salt complexes generally exhibit superior cytotoxicity compared to their neutral counterparts, prompting further exploration of ionic Fe–Ru salt complexes in preference to neutral analogs [120,121,122,123,124]. In addition, prior observations indicated that neutral Fe–Ru complexes were significantly more active than analogous Ru–Ru species, highlighting the potential of heterometallic Fe–Ru systems and providing a rationale for investigating their salt forms. In this study, the authors focused on the effect of the tether ligand’s chain length on the biological activity of these complexes. This led to the synthesis of a series of five mononuclear iron salt complexes and four heterobinuclear Fe–Ru complexes (Figure 19a–d). The mononuclear iron salts were obtained via a one-pot reaction involving the oxidative cleavage of [(η^5^-C_5_H_5_)Fe(CO)_2_]_2_ with [Fe(η^5^-C_5_H_5_)_2_]^+^PF_6_^−^, followed by the addition of the corresponding bis-diphenylphosphine ligand. Subsequently, the heterobimetallic Fe–Ru complexes were synthesized by reacting these mononuclear precursors with [(η^6^-p-cym)RuCl_2_]_2_. The cytotoxic properties of these complexes were evaluated against human ovarian cancer cells (A2780), a cisplatin-resistant variant (A2780cis), and non-cancerous human embryonic kidney cells (HEK293T). The screening results revealed a striking linear correlation between the tether length of the bimetallic complexes and their antineoplastic activity. The authors hypothesized that this trend could arise from one or both of two factors: the longer tether chains may enhance the hydrophobic character of the molecules, promoting cellular uptake, or they may provide increased conformational flexibility between the Fe and Ru centers, which could improve interactions with biological targets. Interestingly, while the bimetallic complexes were active against cancer cells, they also exhibited cytotoxicity toward HEK293T cells. This behavior contrasts with previously studied mononuclear Fe-containing complexes (Figure 17a,b), which showed greater selectivity for malignant cells over healthy ones. To investigate potential mechanisms of cellular uptake, the authors conducted transferrin-binding assays using ESI-MS. The results revealed new spectral signals in the presence of the bimetallic complex (Figure 19c), suggesting potential binding to transferrin. This observation points to a possible transferrin-mediated uptake pathway, a mechanism previously reported for certain ruthenium-containing complexes [125]. Notably, similar experiments using the mononuclear iron precursors did not generate new signals, indicating that heterobimetallic assembly may be crucial for transferrin interaction. Overall, these studies highlight the importance of both the metal composition and ligand architecture in tuning the biological activity of Fe–Ru complexes. The results suggest that careful manipulation of tether length and metal pairing can influence cytotoxicity, selectivity, and potential uptake mechanisms, providing valuable insights for the design of next-generation heterometallic anticancer agents.

**Figure 20:**
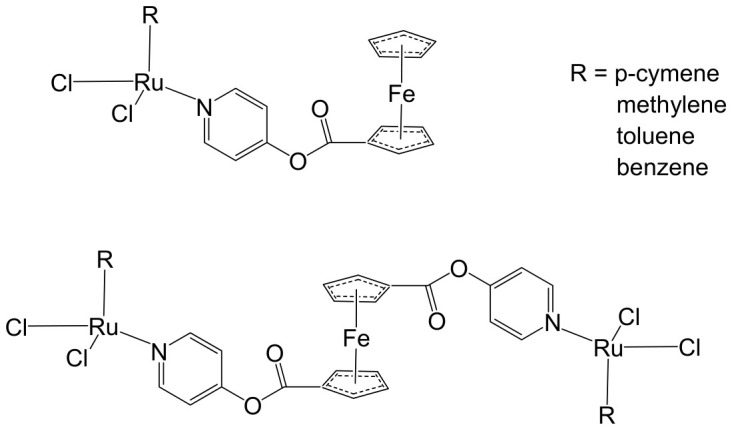
Ferrocenoylpyridine arene ruthenium complexes.

Auzias et al. explored the design of novel antineoplastic agents by combining ruthenium complexes with ferrocene-based ligands (Figure 20) [126]. Their rationale was to exploit the complementary properties of ruthenium, known for its versatile coordination chemistry and relatively low systemic toxicity, and ferrocene, which possesses unique redox characteristics that can influence cellular oxidative stress. In vitro studies using human ovarian cancer cell lines revealed that the ruthenium bimetallic complexes displayed approximately twice the cytotoxic activity of their monometallic counterparts. This enhancement suggests that the Ru–arene motifs play a central role in modulating the complexes’ bioactivity, possibly by facilitating interactions with biomolecular targets or promoting cellular uptake. Interestingly, ruthenium complexes in which the metal centers were linked via simple alkyl chains did not show a comparable increase in cytotoxicity relative to mononuclear analogs [127]. This observation highlights the contribution of the ferrocene moiety’s redox potential to the observed antitumor effects, likely through mechanisms involving reactive oxygen species generation or modulation of cellular redox balance. The IC_50_ values for the bimetallic complexes ranged from 14.8 to 49.5 μM, which are considerably higher than that of the classical chemotherapeutic agent cisplatin (1.6 μM) in the same ovarian cancer cell lines. Nevertheless, these values are noteworthy in the context of ruthenium-based compounds, which generally exhibit lower intrinsic in vitro cytotoxicity. These findings underscore the potential of combining ruthenium centers with redox-active ligands such as ferrocene to enhance antitumor activity, offering a promising avenue for the rational design of next-generation metal-based chemotherapeutics.

**Figure 21:**
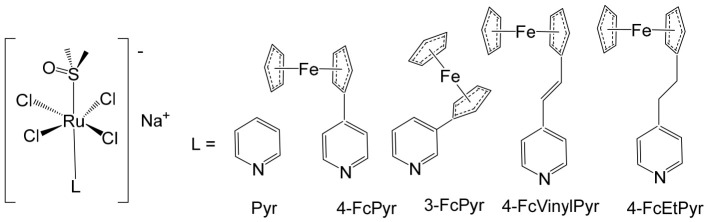
Ferrocene-functionalized NAMI-A analogs.

Building on the notable success of NAMI-A as an antimetastatic agent, Mu et al. [128] explored a novel series of ferrocene-functionalized NAMI-A analogs (Figure 21), aiming to combine the unique properties of ruthenium and ferrocene within a single molecular framework. These studies revealed that both metal centers contribute critically, and in a synergistic manner, to the overall biological profile of the complexes, influencing solubility, cellular uptake, and activity. The coordination of ferrocenylpyridine ligands to the NAMI-A core not only substantially enhanced water solubility but also promoted the activation of the complexes in aqueous media, a key factor for their effective biological performance. In direct comparison with the ferrocene-free analog NAMI-Pyr (IC_50_ > 400 µM), the NAMI-Fc complexes demonstrated significantly higher cytotoxicity against colorectal adenocarcinoma SW480 cells (IC_50_ = 35–69 µM), achieving levels of activity comparable to those of clinically advanced ruthenium(III) agents KP1019 and NKP1339. This enhancement underscores the powerful contribution of the ferrocene unit to the overall therapeutic potential, likely by modulating redox behavior and cellular interactions. Mechanistic research further suggested that the ferrocene moiety facilitates non-coordinate interactions with human serum albumin (HSA) while simultaneously suppressing the formation of protein-bound species. This dual effect improves bioavailability and enables more efficient transmembrane transport, which is essential for reaching intracellular targets. Importantly, the key anti-invasive properties of NAMI-A were preserved: migration assays confirmed that incorporation of ferrocenylpyridine ligands did not compromise the inhibition of cell motility, a critical determinant of antimetastatic efficacy. Taken together, these findings illustrate that the NAMI-Fc complexes achieve a rare combination of antimetastatic and cytotoxic activities. They highlight how strategic incorporation of the ferrocene unit can enhance pharmacological properties without sacrificing the parent compound’s beneficial effects, providing a promising blueprint for the rational design of next-generation ruthenium-based therapeutics.

## 6. Ruthenium–Iridium Complexes

Cyclometallated Ir(III) complexes are distinguished by their exceptional optical properties and have been widely applied in cellular imaging and biomedical research [129,130]. Their strong luminescence, coupled with remarkable stability in complex biological environments, positions them as highly effective emissive tags for tracking therapeutic agents and probing cellular processes. Beyond imaging, many cyclometallated Ir(III) complexes exhibit potent activity as photosensitizers in photodynamic therapy [131], highlighting their potential as multifunctional agents that bridge diagnostics and therapy. The versatility of these complexes opens avenues for the development of next-generation theranostic platforms, combining real-time imaging, targeted treatment, and controlled therapeutic delivery in a single molecular framework.

**Figure 22:**
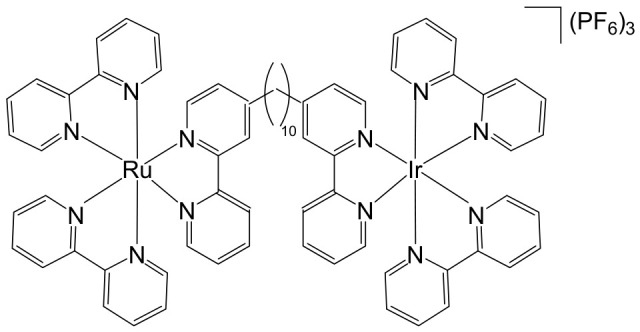
The structure of the heterodinuclear Ir(III)–Ru(II) complex [Ru(bpy)_3_-(CH_2_)_10_-Ir(F_2_ppy)_2_]^3+^.

Studies by Sun et al. [132] have explored heterodinuclear Ir(III)–Ru(II) complex [Ru(bpy)_3_-(CH_2_)_10_-Ir(F_2_ppy)_2_]^3+^ (ppy = 1-phenyl-pyridine; bpy = 2,2′-bipyridine), Figure 22, demonstrating that such heterobimetallic systems possess substantial binding affinities toward both DNA and rRNA. The complex, illustrated in Figure 22, exhibits dual photoluminescence in the green (523 nm) and orange (615 nm) regions, a property that can be harnessed for multichannel bioimaging. Colocalization studies further confirmed its selective accumulation in the nucleolus, suggesting a pronounced affinity for RNA over DNA. This combination of strong nucleic acid binding and dual-emission characteristics highlights the complex’s potential not only as a sophisticated imaging probe but also as a candidate for RNA-targeted therapeutic strategies. Such heterobimetallic complexes thus represent a promising platform for the development of multifunctional agents in chemical biology and medicinal chemistry.

**Figure 23:**
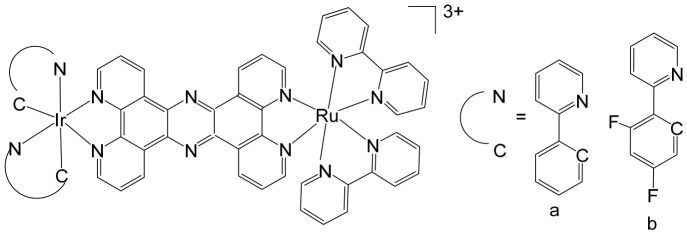
Dinuclear tpphz complexes [Ir(ppy)_2_(tpphz)Ru-(bipy)_2_]^3+^ and [(F_2_ppy)_2_lr(tpphz)Ru(bipy) _2_]^3+^, where N^C is 2-phenyl-pyridine (**a**) and 2-(4-fluorophenyl)pyridine (**b**).

Wragg et al. have synthesized a series of dinuclear tpphz complexes incorporating both iridium(III) and ruthenium(II) centers, specifically designed for applications in cellular imaging with the general formula [Ir(ppy)_2_(tpphz)Ru-(bipy)_2_]^3+^ and [(F_2_ppy)_2_lr(tpphz)Ru(bipy)_2_]^3+^ (ppy = 2-phenyl-pyridine and F_2_ppy = 2-(4-fluorophenyl)pyridine). These complexes demonstrate preferential localization in the cell nucleus, as observed for the complexes (Figure 23) [133]. Notably, the incorporation of the iridium(III) component significantly enhanced cellular uptake when compared to the homonuclear Ru–Ru complex bearing bipyridine ligands. This enhancement is believed to arise from a combination of factors, including the lower overall positive charge of the heteronuclear complex and the increased lipophilicity imparted by the iridium(III) center and its associated ligands, which together facilitate more efficient passage through the cellular membrane. In addition, the substitution of bipyridine ligands with more lipophilic phenanthroline ligands in these dinuclear tpphz complexes further improved cellular accumulation. Importantly, this modification did not compromise the DNA-binding capability of the complexes, suggesting that careful tuning of ligand properties can optimize uptake without affecting the primary biological target. This strategy of enhancing lipophilicity has been widely employed in studies of ruthenium complexes to promote passive diffusion into cells; however, it carries the risk of off-target interactions. Specifically, highly lipophilic compounds may bind to alternative intracellular structures, such as membranes or the endoplasmic reticulum, potentially diverting them from their intended nuclear target [134]. Despite these considerations, the dinuclear complexes described here exhibit both good water solubility and strong affinity for DNA, making them suitable for nuclear-targeted imaging. Cellular experiments confirmed effective nuclear uptake, and this effect was particularly pronounced in fluorinated derivatives, which targeted the nucleus more rapidly than their non-fluorinated counterparts. Overall, these findings highlight the importance of balancing lipophilicity, charge, and ligand design in the development of metal-based imaging agents, enabling efficient nuclear localization while minimizing unintended interactions elsewhere in the cell.

**Figure 24:**
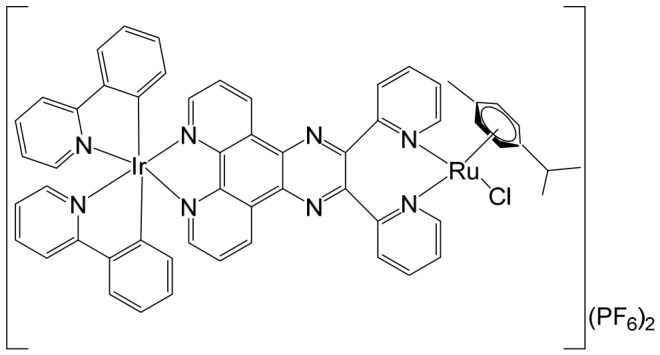
Structure of dinuclear Ir–Ru complex [{(ppy)_2_Ir}(µphpy){Ru(p-cym)Cl}](PF_6_)_2_ with pyrazino [2,3-f][1,10]phenanthroline ligand.

Cyclometallated Ir(III) complexes of the general formula [Ir(N^C)_2_(N^N)]^0^/^+^, where N^C denotes an orthometallated ligand and N^N a neutral diimine chelator, have attracted considerable attention in medicinal inorganic chemistry. Their straightforward synthetic accessibility, structural tunability, photophysical versatility, and notable stability toward air and moisture make them highly appealing scaffolds for the development of heterometallic theranostic systems. In particular, the combination of luminescent Ir(III) fragments with bioactive metal centers enables the integration of diagnostic and therapeutic functions within a single molecular framework. Within this context, Tripathy et al. described the design, synthesis, and biological evaluation of a heterometallic dinuclear Ir–Ru complex, [{(ppy)_2_Ir}(µ-phpy){Ru(p-cym)Cl}](PF_6_)_2_, in which 2-phenylpyridine (ppy) acts as the cyclometallating ligand and pyrazino [2,3-f][1,10]phenanthroline (phpy) serves as a bridging polypyridyl ligand between the two metal centers. Structurally, the Ir(III) moiety adopts a distorted octahedral geometry typical of cyclometallated Ir(III) systems, whereas the Ru(II) center exhibits the characteristic η^6^-arene “piano–stool” arrangement (Figure 24) [135]. The rationale behind this heterometallic design was to identify agents capable of inducing autophagy rather than apoptosis, thereby offering a strategy to overcome resistance mechanisms frequently encountered in conventional chemotherapy. Autophagy-inducing compounds are of particular interest because they can selectively target tumor cells that evade apoptosis. The authors hypothesized that combining two metal fragments within a single polymetallic framework would provide synergistic biological effects. Moreover, the incorporation of extended polypyridyl ligands was expected to enhance lipophilicity, cellular uptake, and potential DNA or biomolecular interactions [136]. Biological evaluation revealed that the Ir–Ru complex displayed enhanced cytotoxic activity compared with its homonuclear diruthenium analog across multiple human cancer cell lines, including breast (MCF7), ovarian (SKOV3), prostate (PC3), and endometrial (Ishikawa) cells. Particularly noteworthy was its potency in cisplatin-resistant MCF7 cells, with an IC_50_ value of 0.92 µM, highlighting its potential to circumvent platinum resistance. To elucidate the mechanism of action, cell cycle distribution was analyzed by flow cytometry, revealing arrest in the G1 phase. Western blot studies were conducted to assess the expression levels of apoptosis-related proteins, including the anti-apoptotic Bcl-2, the pro-apoptotic Bax, and the cleavage of poly(ADP-ribose) polymerase (PARP), a central mediator of DNA repair and apoptotic signaling [137]. The absence of significant changes in these markers indicated that the observed cytotoxicity was not mediated by classical apoptotic pathways. Further morphological analysis demonstrated extensive cytoplasmic vacuolization, a characteristic feature of autophagic cell death [138]. Collectively, these findings establish the complex [{(ppy)_2_Ir}(µ-phpy){Ru(p-cym)Cl}](PF_6_)_2_ (Figure 24) as the first reported heteronuclear Ir–Ru complex capable of inducing autophagy in cisplatin-resistant breast cancer cells. The designed heterometallic system combines the photophysical advantages of cyclometalated Ir(III) fragments with the established bioactivity of Ru(II) arene units. Nevertheless, comprehensive mechanistic investigations, particularly at the molecular and intracellular levels, are still required to fully clarify the pathways involved and to assess the translational potential of this class of compounds.

**Figure 25:**
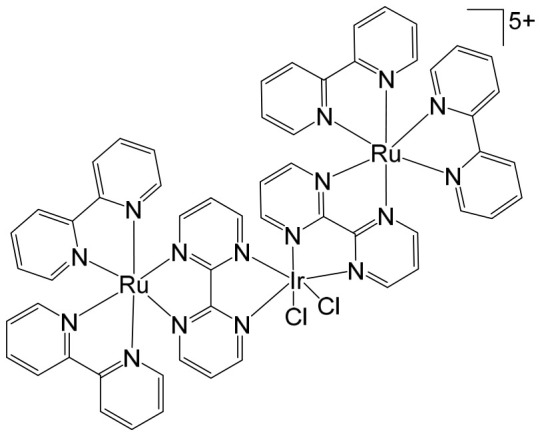
Structure of the trimetallic complex {[(bpy)_2_Ru(bpm)]_2_IrCl_2_}^5+^.

Nallas et al. described the synthesis and in-depth characterization of a bipyrimidine-bridged trimetallic complex {[(bpy)_2_Ru(bpm)]_2_IrCl_2_}^5+^ (Figure 25), where bpy = 2,2′-bipyridine and bpm = 2,2′-bipyrimidine [139]. In this molecular architecture, two photoactive Ru(II) polypyridyl chromophores are covalently linked to a central Ir(III) unit through bridging bpm ligands, generating a discrete polymetallic assembly in which light absorption and catalytic functionality are structurally integrated. Comprehensive spectroscopic, electrochemical, and spectroelectrochemical analyses revealed strong electronic communication across the metal centers and ligand framework. On the basis of these findings, the authors proposed that such systems can operate as molecular photochemical devices, capitalizing on the light-harvesting properties and long-lived excited states of Ru(II) in conjunction with the catalytic competence of the Ir(III) center [140]. Extending this design strategy, the same group explored a broader family of heterodimetallic and higher-order polymetallic complexes of the general formula {[(bpy)_2_Ru(BL)]_2_MCl_2_}^n+^, where BL denotes π-extended bridging ligands such as 2,3-bis(2-pyridyl)pyrazine (dpp), 2,3-bis(2-pyridyl)quinoxaline (dpq), and 2,3-bis(2-pyridyl)benzoquinoxaline (dpb), and M = Ir(III), Rh(III), or Os(II) [141,142]. Systematic variation of both the bridging ligand and the secondary metal center enabled fine control over metal-to-ligand charge-transfer (MLCT) energies, redox potentials, and excited state lifetimes. These studies demonstrated that increasing ligand conjugation and altering the identity of the ancillary metal significantly influence intramolecular charge separation and recombination dynamics. Collectively, this work underscores the broader versatility of extended Ru-based supramolecular assemblies as tunable platforms for directing charge transfer processes and optimizing catalytic behavior in light-driven applications.

**Figure 26:**
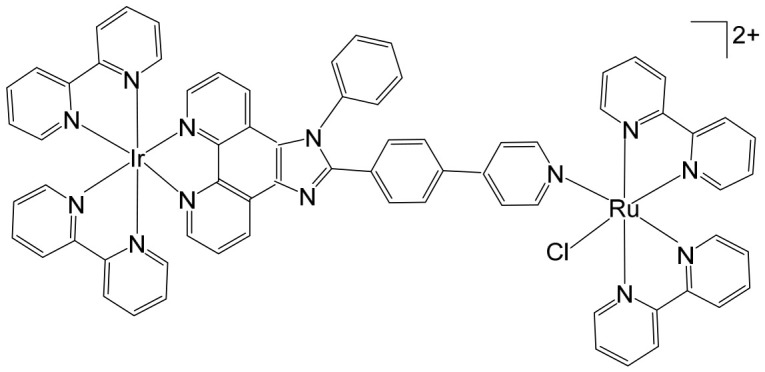
The structure of the complex {[(bpy)_2_Ir(dpip)]Ru(bpy)_2_Cl_2_}^2+^.

Zhang et al. [143] described the synthesis and biological evaluation of a heterobimetallic ruthenium(II)–iridium(III) complex {[(bpy)_2_Ir(dpip)]Ru(bpy)_2_Cl_2_}^2+^ rationally engineered as a bifunctional platform for the integration of photoactivated chemotherapy and photodynamic therapy (Figure 26). The complex has been synthesized by the interaction of [(ppy)_2_Ir(μ-Cl)]_2_, 1,2-diphenyl-1H-imidazo [4,5-f][1,10]phenanthroline (dpip), and Ru(bpy)_2_Cl_2_. The design concept strategically combined the complementary properties of both metal centers: the cyclometalated Ir(III) unit was incorporated to promote selective mitochondrial localization, while the Ru(II) fragment was introduced to facilitate mitochondrial DNA (mtDNA) binding and damage. This cooperative architecture was intended to exploit mitochondrial vulnerability and amplify therapeutic efficacy through dual mechanistic pathways. Biological studies revealed that the complex displayed pronounced antineoplastic activity across a panel of cancer cell lines, including those resistant to cisplatin, indicating its potential to overcome conventional platinum-based drug resistance. Cellular uptake and localization experiments confirmed preferential accumulation within mitochondria, consistent with the targeting role of the Ir(III) moiety. A particularly notable feature of this system is its controlled photochemical behavior. The complex generated singlet oxygen (^1^O_2_) exclusively under sequential irradiation conditions, initial excitation at 450 nm followed by secondary irradiation at 405 nm, demonstrating a stepwise activation mechanism. This dual-wavelength requirement provides a higher level of spatial and temporal control over reactive oxygen species (ROS) production, potentially minimizing off-target phototoxicity in non-irradiated tissues. Functionally, the heterobimetallic construct induced substantial mitochondrial dysfunction. Treated cells exhibited a significant decrease in mitochondrial membrane potential (MMP), reflecting compromised mitochondrial integrity. This depolarization event was accompanied by activation of the intrinsic apoptotic pathway, ultimately leading to programmed cell death. Collectively, these findings underscore the therapeutic relevance of this dual-functional, mitochondria-targeted design, which integrates organelle-specific DNA damage with precisely controlled photodynamic activity to enhance anticancer performance.

**Figure 27:**
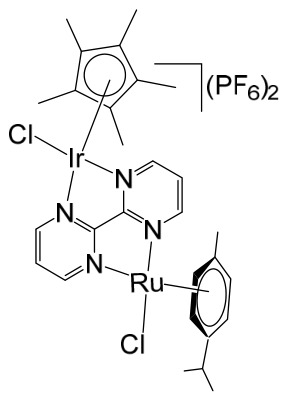
The structure of heterobimetallic complex [Ir(η^5^-Cp*)Cl(μ-bpm)Ru(η^6^-p-cym)Cl](PF_6_)_2_.

Recently, luminescent ruthenium(II)–iridium(III) complexes incorporating the 2,2′-bipyrimidine (bpm) ligand have been synthesized and characterized [144]. Among these, the monochlorido Ir–Ru complex [Ir(η^5^-Cp*)Cl(μ-bpm)Ru(η^6^-pcym)Cl](PF_6_)_2_ (Figure 27), featuring a tetradentate bpm bridging ligand, was investigated alongside its diruthenium and diiridium analogs in breast (MDA-MB-468) and colon (Caco-2) cancer cell lines. In breast cancer cells, the heterometallic Ru–Ir complex displayed moderate cytotoxic activity (IC_50_ = 1.9 μM), with potency comparable to that of the diiridium analog (IC_50_ = 1.8 μM) but lower than that of the diruthenium complex (IC_50_ = 0.9 μM). By contrast, in colon cancer cells, the Ru–Ir compound demonstrated a markedly enhanced antiproliferative effect (IC_50_ = 6.2 μM), significantly outperforming both homometallic counterparts (IC_50_ = 32.4 μM for the diiridium and 46.0 μM for the diruthenium complex). Notably, the heterometallic system also proved substantially more active than its corresponding mononuclear precursors, [Ir(η^5^-Cp*)(bpm)Cl]PF_6_ (IC_50_ = 50.4 μM) and [Ru(η^6^-pcym)(bpm)Cl]PF_6_ (IC_50_ = 49.6 μM), underscoring the beneficial effect of metal–metal combination within a single molecular framework. Taken together, these results demonstrate that incorporation of both ruthenium and iridium centers within a bpm-bridged architecture can lead to synergistic enhancement of cytotoxic properties, particularly against colon cancer cells. This highlights the potential of heterometallic design strategies as a promising approach for the development of more effective anticancer metallodrugs.

## 7. Ruthenium–Palladium Complexes

Palladium complexes are closely related to their platinum analogs owing to their similar physicochemical characteristics. Like platinum, palladium is utilized in antitumor agents in the Pd^2+^ and Pd^4+^ oxidation states, and these similarities permit their incorporation into structurally analogous anticancer compounds [145]. Both metals belong to the platinum-group elements (PGE) and exhibit comparable coordination geometries and metal–ligand bond lengths. Despite these parallels, important differences distinguish their chemical behavior. Palladium complexes undergo ligand substitution reactions approximately 10^5^ times faster than platinum complexes, which necessitates stabilization through the use of strong chelating ligands to enhance kinetic stability under physiological conditions. This disparity arises primarily from their electronic configurations: Pd ([Kr]4d^10^) and Pt ([Xe]4f^14^5d^9^6s^1^). Platinum’s electronic structure, particularly the involvement of its 5d orbitals, results in a higher ionization potential and stronger metal–ligand bonding. Furthermore, the relatively small energy gap between the 5d and 6s orbitals in platinum contributes to additional thermodynamic stability of its complexes. From a biological perspective, toxicological studies suggest that palladium compounds are approximately ten times less toxic than platinum-based drugs [145]. While platinum cytostatic agents remain central to chemotherapy, their clinical use is associated with several limitations, including poor water solubility, dose-limiting toxicity, and the development of intrinsic or acquired resistance in cancer cells. These drawbacks have intensified efforts to explore palladium-based systems as promising alternatives. In particular, the design of ruthenium–palladium heterometallic complexes represents an emerging strategy aimed at combining favorable pharmacological properties of both metals, thereby opening broader prospects for the development of more effective and potentially less toxic anticancer therapies.

**Figure 28:**
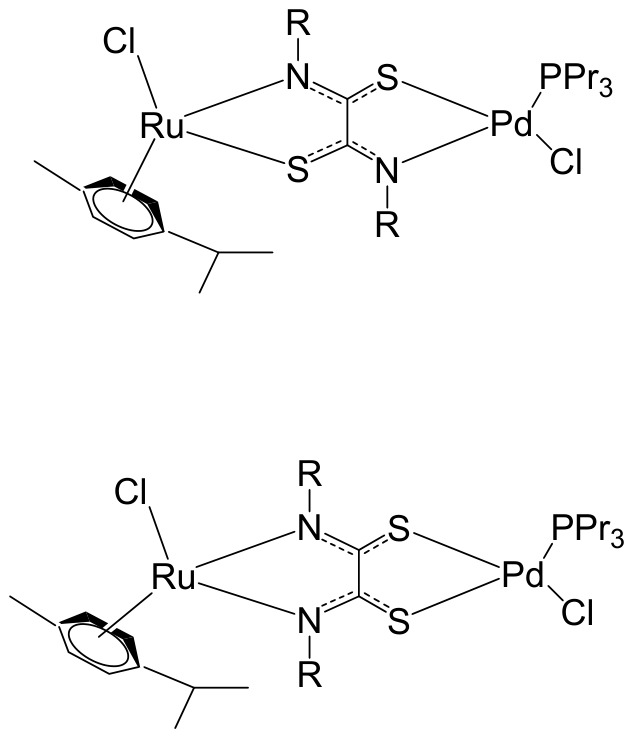
Ruthenium–palladium heterobimetallic complexes, where R = methyl (a), ethyl (b), n-butyl (c) and isopropyl (d).

Ruthenium–palladium heterobimetallic complexes (Figure 28) were synthesized in two distinct coordination modes, NNSS and NS, using a binucleating dialkyldithiooxamidate ligand [N(R)SC-CS(R)N], where R represents methyl, ethyl, n-butyl, or isopropyl groups. This ligand effectively bridges the ruthenium and palladium centers, enabling the formation of structurally well-defined heterobimetallic species. The complexes were prepared through the reaction of the monochelate precursor [(tri*^n^*propyl-phosphine)ClPd(HR_2_C_2_N_2_S_2_ κ-S,S-Pd)] with the ruthenium dimer [(η^6^-*p*-cymene)RuCl_2_]_2_. During these studies, it was observed that the NNSS coordination mode was kinetically accessible but thermodynamically unstable, leading to spontaneous rearrangement into the more stable NS coordination mode [146]. Detailed structural characterization, including X-ray crystallography, confirmed the geometry of the heterobimetallic complexes, and computational studies provided additional insights into their electronic structure and relative stabilities. Once isolated, the stable NS complexes were evaluated for their biological activity against leukemia cell lines. Specifically, they were tested on the drug-sensitive CCRF-CEM cells and the multidrug-resistant CEM/ADR5000 subline. The results revealed that all NS complexes exhibited significant antiproliferative activity, with IC_50_ values in the low micromolar range (approximately 1–5 μM), highlighting their potential as cytotoxic agents. Moreover, these heterobimetallic complexes demonstrated an ability to inhibit the proteolytic function of the tumor-associated 20S proteasome, which is a validated anticancer target. Most complexes were capable of blocking at least two out of the three main proteolytic activities, with the complex depicted in Figure 28 showing the most pronounced inhibitory profile. These findings suggest that the combination of Ru and Pd within a single molecular framework, coupled with the versatile dialkyldithiooxamidate bridging ligand, confers both structural stability and significant biological activity, making these complexes promising candidates for further anticancer investigations.

**Figure 29:**
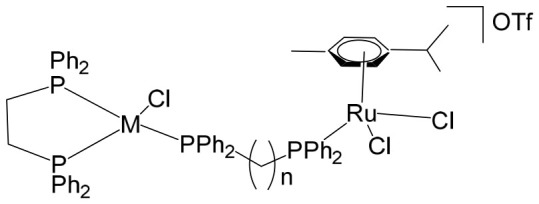
Heterodinuclear Pd(II)–Ru(II) complexes, where n = 4 (a), n = 5 (b), n = 6 (c) and Pt(II)–Ru(II) complexes, where n = 4 (d), n = 5 (e), n = 6 (f).

Heterobinuclear Pd(II)–Ru(II)- and Pt(II)–Ru(II)-based complexes [(κ^2^-dppe)ClM(μ-dppx)RuCl_2_(η^6^-*p*-cym)](OTf) (M = Pd and dppx = dppb, dpppent, and dpph, M = Pt and dppx = dppb, dpppent, and dpph) have been recently reported [147]. The complexes (Figure 29) were fully characterized using spectroscopic techniques, and their stability in DMSO was assessed through time-dependent NMR spectroscopy [147]. The design of this series of complexes addresses multiple aspects of drug development. First, analogous complexes differing only in the central transition metal (i.e., Pd(II) vs. Pt(II) vs. Ru(II)) were evaluated to determine which metal imparts the highest activity. Second, consistent with multiple previous studies, monometallic complexes generally exhibit lower activity than bimetallic complexes, and homobimetallic complexes are often less active than heterobimetallic counterparts. Structurally, the complexes exhibit a distorted square-planar geometry at the Pd(II) and Pt(II) centers. In contrast, the Ru(II) center adopts a distorted octahedral geometry, with the η^6^-p-cymene ligand occupying three coordination sites and the remaining ligands occupying the other three positions. This “piano–stool” configuration is characteristic of Ru(II) complexes, and numerous examples of [(*p*-cym)RuCl_2_(PPh_2_R)] complexes have been reported. Cell viability was systematically evaluated across eight human cancer cell lines, encompassing cervical (HeLa and Ca Ski), pancreatic (PANC-1 and CFPAC-1), rhabdomyosarcoma (RD and RH30), and breast (MCF-7 and MDA-MB-231) cancers. To assess selectivity toward malignant versus non-malignant cells, the most active compounds were also tested on the non-malignant MRC-5 cell line. For the most potent complexes, IC_50_ values were determined in cervical and pancreatic cancer cell lines, and selectivity indices were calculated by cross-testing on FG0 non-malignant cells, providing a quantitative measure of therapeutic window. Analysis of structure–activity relationships revealed no obvious correlation between the diphosphine tether or spacer length and biological activity. In contrast, a pronounced link was observed between the heterobimetallic composition of the complexes and enhanced cytotoxicity. This effect is likely due to the formation of distinct aquated species in biological media, as suggested by preliminary aquation studies, which may modulate cellular uptake, reactivity, and target interactions. In comparison, monometallic and homobimetallic analogs displayed suboptimal in vitro activities, emphasizing that heterometallic frameworks may be critical for achieving superior anticancer effects, particularly for complexes containing Pd(II) and Pt(II). Taken together, the data highlight a novel class of heterobimetallic complexes that exhibit promising cytotoxic profiles and selectivity. These findings underscore the potential of heterobimetallic Pd(II)–Ru(II) and Pt(II)–Ru(II) scaffolds as an innovative design strategy for next-generation anticancer agents, providing a rationale for further optimization and mechanistic studies to explore their therapeutic potential and mode of action.

## 8. Ruthenium–Osmium Complexes

Ruthenium and osmium, situated next to each other in the periodic table, exhibit strikingly similar coordination chemistry, yet their biological and redox behaviors reveal fascinating divergences. Ruthenium(II) complexes have long been studied for their remarkable kinetic stability and their ability to mimic iron in biological systems, which allows them to interact effectively with biomolecules such as DNA and proteins. This iron-mimicking behavior often translates into pronounced cytotoxic effects, making Ru(II) complexes promising candidates for anticancer and other therapeutic applications. Their well-defined ligand exchange kinetics and predictable coordination environments further enhance their utility in the design of biologically active compounds. In contrast, osmium(II) complexes, while chemically akin to their ruthenium counterparts, have been less extensively explored in biological contexts. They exhibit notably slower ligand exchange rates, which can lead to increased kinetic inertness under physiological conditions. At the same time, osmium complexes often display enhanced redox versatility, enabling access to multiple oxidation states and offering the potential for finely tuned modulation of biological activity. These properties make Os(II) complexes particularly attractive for applications where controlled reactivity and redox activity are desired, although their full biological potential remains to be systematically investigated [148].

**Figure 30:**
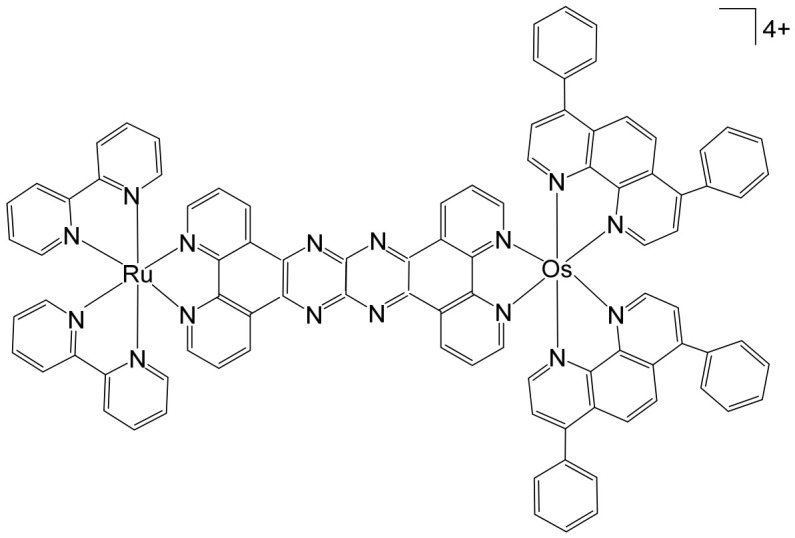
The heteronuclear [Ru(bpy)_2_(pppp)Os(dip)_2_](PF_6_)_4_ complex.

Heterodinuclear ruthenium–osmium complexes have been successfully synthesized by reacting mononuclear modules derived from homodinuclear Ru–Ru complexes, which possess dual photodynamic therapy (PDT) and photothermal therapy (PTT) activity, with Os–Os complexes that exhibit strong PTT activity but lack PDT capability [149]. The heteronuclear [Ru(bpy)_2_(pppp)Os(dip)_2_](PF_6_)_4_ (Figure 30), where bpy = 2,2′-bipyridine, dip = 4,7-diphenyl-1,10-phenanthroline, has been prepared by the interaction of the ligand pyrazino [2,3-f][1,10]phenanthroline-2,3-diamine with [Ru(bpy)_2_(phendione)]. Further the mononuclear [Ru(bpy)_2_(pppp)](PF_6_)_2_ precursor has been stirred with cis-[Os(dip)_2_Cl_2_] and upon addition of NH_4_PF_6_ the heteronuclear Ru(II)–Os(II) complex [Ru(bpy)_2_(pppp)Os(dip)_2_](PF_6_)_4_ has been obtained. The resulting ruthenium–osmium complex, shown in Figure 30, demonstrates significantly enhanced therapeutic performance compared to its Ru–Ru and Os–Os parent complexes, benefiting from the combined advantages of both metal centers. Mechanistic studies revealed that this complex enters cells predominantly via caveolar endocytosis and selectively accumulates in mitochondria, while avoiding the nucleus. Once localized in mitochondria, it produces singlet oxygen, triggering apoptosis in targeted cancer cells. In vivo experiments in mice showed remarkable efficacy, with complete eradication of PDT-resistant melanoma tumors and cisplatin-resistant non-small cell lung tumors. Importantly, analysis of the mouse organs after treatment revealed only minimal residual metal content, indicating efficient clearance. The complex also exhibited extremely low toxicity toward normal liver and kidney cells, highlighting its potential as a safe and effective dual PDT/PTT anticancer agent.

The synthesis, detailed photophysical characterization, electrochemical investigation, and intramolecular energy transfer properties of two distinct series of dinuclear and tetranuclear complexes—namely, [(bpy)_2_M_1_L_x_M_2_(bpy)_2_]^4+^ (x = 1, 2; M_1_ = Ru, M_2_ = Ru or Os; M_1_ = Os, M_2_ = Ru) and {[Ru(bpy)_2_(L_x_)]_3_Ru}^8+^—constructed using newly designed heteroditopic bridging ligands (L_1_ 6-phenyl-4-Hpip-2-2′-bipyridine, L_2_ = 6-Hpip-2-2′-bipyridine, with Hpip = 2-phenyl-1*H*-imidazo [4,5-*f*][1,10]phenanthroline) have recently been reported [150]. Both the dimetallic and tetranuclear complexes demonstrate remarkably rich redox behavior, characterized by multiple successive and reversible metal-centered oxidation processes alongside ligand-centered reduction couples, highlighting their potential for multielectron charge transfer applications. All complexes exhibit intense absorption spanning the ultraviolet to visible spectral regions, indicative of strong light-harvesting capabilities. In particular, the mononuclear [L_x_Ru(bpy)_2_]^2+^ complexes, as well as the homodinuclear [(bpy)_2_RuL_x_Ru(bpy)_2_]^4+^ analogs, display pronounced Ru^2+^-centered emission at ambient temperature, consistent with their well-known photophysical signatures. Of notable interest, the heterodinuclear complexes [(bpy)_2_M_1_L_x_M_2_(bpy)_2_]^4+^ show almost complete quenching of the Ru^2+^-based emission, accompanied by highly efficient photoinduced energy transfer that preferentially channels excitation energy toward Os^2+^ subunits, giving rise to near-infrared emission characteristic of Os^2+^. In the tetranuclear systems, the combination of intramolecular energy transfer from the central core to the peripheral metal centers and steric hindrance effects, leading to quenching of the peripheral Ru^2+^-centered emissive triplet metal-to-ligand charge transfer states, results in overall weak Ru^2+^-based emission with substantially shortened lifetimes. Because these multinuclear architectures effectively direct the energy absorbed by multiple chromophoric units toward the subunit with the lowest-energy excited state, they are highly promising as visible-light-absorbing antenna systems, capable of efficiently harvesting and funneling light energy for potential applications in photochemical and optoelectronic devices.

## 9. Conclusions and Perspectives

A promising and increasingly explored strategy in anticancer drug design involves the integration of multiple metal centers within ruthenium-based complexes to enhance therapeutic performance while mitigating toxicity and the development of drug resistance. By combining ruthenium with platinum or other non-platinum metals, researchers aim to exploit synergistic or cooperative effects that may overcome the well-documented limitations of monometallic agents, such as limited selectivity, systemic toxicity, and resistance mechanisms. This heterometallic approach builds on the unique biochemical versatility of ruthenium while incorporating complementary properties from additional metal centers. Recent studies have highlighted the significant antineoplastic potential of multinuclear and heteronuclear ruthenium complexes, with numerous compounds demonstrating encouraging in vitro cytotoxicity across diverse cancer cell lines. Unlike traditional single-metal systems, heterometallic ruthenium complexes can engage multiple and distinct biological targets simultaneously, including DNA, proteins, enzymes, redox pathways, and mitochondrial functions. Such multitargeting capacity may produce synergistic cytotoxic effects, reduce the likelihood of acquired resistance, and broaden the spectrum of activity against refractory tumors. Beyond biological activity, the rational incorporation of two or more metals with complementary pharmacological and coordination properties can enhance physicochemical characteristics such as stability, solubility, lipophilicity, and redox behavior. These modifications may improve cellular uptake, biodistribution, tumor selectivity, and controlled activation within the tumor microenvironment. In many cases, heterometallic systems outperform either their monometallic counterparts or simple mixtures of individual metal complexes, suggesting that this integration within a single molecular framework provides distinct therapeutic advantages. Despite these promising developments, mechanistic studies on heterometallic ruthenium complexes remain relatively scarce and frequently preliminary. Comprehensive investigations into their modes of action, intracellular trafficking, target specificity, and long-term biological effects are essential for rational optimization. A deeper understanding of structure–activity relationships and metal–metal cooperativity will be critical to improving selectivity and minimizing off-target toxicity. Furthermore, the catalytic, redox-active, and photophysical properties of certain metal centers introduce exciting possibilities for theranostic applications. Heterometallic ruthenium systems may serve not only as cytotoxic agents but also as diagnostic tools, enabling live-cell imaging, real-time tracking, or stimulus-responsive activation. Such multifunctional behavior is largely inaccessible to conventional metallodrugs and represents a significant advantage of heteronuclear designs. Although many heteronuclear ruthenium-based compounds exhibit multitargeting properties and promising in vitro anticancer activity, the limited availability of robust in vivo data has constrained their clinical translation. Comprehensive preclinical evaluation, including pharmacokinetics, biodistribution, metabolism, toxicity, and therapeutic efficacy in animal models, is urgently needed to validate their potential and ensure safety. Overall, heteronuclear ruthenium complexes represent a dynamic and expanding frontier in anticancer drug development. Their structural versatility, multitargeting capabilities, and potential for theranostic integration position them as compelling candidates for next-generation metallodrugs. Continued interdisciplinary research combining synthetic chemistry, molecular biology, pharmacology, and translational studies is essential to fully harness their capabilities. This review provides a broad overview of current advances in the medicinal chemistry of heterometallic ruthenium complexes with anticancer potential and outlines future directions for the rational design of innovative metal-based therapeutics in oncology.

## Data Availability

No new data were created or analyzed in this study.
